# *MALE STERILE6021* (*MS6021*) is required for the development of anther cuticle and pollen exine in maize

**DOI:** 10.1038/s41598-017-16930-0

**Published:** 2017-12-01

**Authors:** Youhui Tian, Senlin Xiao, Juan Liu, Yamuna Somaratne, Hua Zhang, Mingming Wang, Huairen Zhang, Li Zhao, Huabang Chen

**Affiliations:** State Key Laboratory of Plant Cell and Chromosome Engineering, Institute of Genetics and Developmental Biology, Chinese Academy of Sciences, Beijing, 100101 China

## Abstract

The anther cuticle and pollen wall function as physical barriers that protect genetic material from various environmental stresses. The anther cuticle is composed of wax and cutin, the pollen wall includes exine and intine, and the components of the outer exine are collectively called sporopollenin. Other than cuticle wax, cutin and sporopollenin are biopolymers compounds. The precise constituents and developmental mechanism of these biopolymeric are poorly understood. Here, we reported a complete male sterile mutant, *male sterile6021*, in maize. The mutant displayed a smooth anther surface and irregular pollen wall formation before anthesis, and its tapetum was degraded immaturely. Gas chromatography-mass spectrometry analysis revealed a severe reduction of lipid derivatives in the mutant anther. We cloned the gene by map based cloning. It encoded a fatty acyl carrier protein reductase that was localized in plastids. Expression analysis indicated that *MS6021* was mainly expressed in the tapetum and microspore after the microspore was released from the tetrad. Functional complementation of the orthologous *Arabidopsis* mutant demonstrated that *MS6021* is conserved between monocots and dicots and potentially even in flowering plants. *MS6021* plays a conserved, essential role in the successful development of anther cuticle and pollen exine in maize.

## Introduction

In flowering plants, male reproductive development is essential for metagenesis and genetic recombination, which is also a complex process in which cooperative interactions occur between sporophytic and gametophytic tissues^1,2^. After anther morphogenesis, each anther locule includes centrally localized pollen mother cells (PMC) surrounded by four somatic layers, from the surface to the interior: the epidermis (E), endothecium (En), middle layer (ML), and tapetum (T)^2–4^. As a secretory cell layer, the tapetum provides abundant ingredients for the anther cuticle and pollen outer wall^5,6^. These two rigid barriers protect the genetic material in microspores or pollen grains from various biotic and abiotic stresses^7,8^.

The anther cuticle is located outside of the epidermis. It seals plant anther against the environment. As the skin of the anther, the cuticle is mainly composed of cutin and cuticle wax. Cuticle wax impregnates or covers cutin^9–11^. Hydrophobic cutin is a polymer of hydroxylated and epoxylated fatty acids and their derivatives with chain lengths of C16 and C18^12^. Cuticle wax is composed of very long-chain fatty acids (VLCFA), alkanes, alkene, and fatty alcohols, among others^9^. The pollen wall is a multilayer, robust structure surrounding the pollen cytoplasm. The outer layer, called the exine, is principally composed of sporopollenin, highly resistant biopolymers derived from fatty acids, phenylpropanoids, and phenolic^13^. Although sporopollenin is commonly present in pollen grains and spores^14^, the fine structure of the exine is varies among species^15^. The durability of the exine combined with its species-specific structure enable its application in paleontological and forensic analyses^16^. However, the understanding of the biochemical components and biosynthesis of the exine remains largely elusive due to its high insolubility and chemical resistance.

Recent genetic and biochemical investigations of the development of *Arabidopsis* and rice anthers have greatly facilitated our understanding of the synthesis regulation of aliphatic biopolymers, such as anther cuticle and sporopollenin^17^. *Arabidopsis MALE STERILITY 2* (*MS2*)^18^ and rice *DEFECTIVE POLLEN WALL* (*DPW*)^19^ in plastids catalyze the reduction of fatty acyl-ACP to fatty alcohols. *CYP703A2* and *CYP703A3* ^20^ function as lauric acid hydrolxylase^21^. *CYP704B1* ^22^ and *CYP704B2* ^7^ catalyze the ω-hydroxylation of fatty acid. Both *CYP703A*s and *CYP704B*s belong to the ancient and conserved P450 gene family^23^. *ACYL COENZYME A SYNTHETASE 5* ^24^, two *POLYKETIDE SYNTHESES*, *PKSA/LAP6* and *PKSB/LAP5* ^25,26^, *TETRAKETIDE α-PYRONE REDUCTASE 1* (*TKPR1*)^27,28^ are proposed to function together in the synthesis of hydroxylated tetraketide α-pyrones, which are polyketides that may form the major constituent of sporopollenin^29^. All the above mentioned genes related to lipid-soluble precursor synthesis are predominantly expressed in tapetal cells. After the biosynthesis steps, these precursors must be secreted from the tapetum and transferred to the outside surface of microspores and anther wall surfaces to be polymerized into biopolymers of sporopollenin, and cutin^30^ respectively. According to recent investigations, ATP-binding cassette (ABC), lipid transfer protein (LTP), and multidrug and toxic efflux (MATE) protein may be responsible for the transport of biopolymer precursors^23^. *OsABCG15* is believed to transfer lipid monomers for anther cuticle and exine development^31^, while its ortholog, *AtABCG26*, transports both lipid precursors and polyketides for exine formation^29,32^. *OsC6* encodes a lipid transfer protein. It is speculated to transfer lipidic molecules from tapetal cells to other anther cells and pollen wall surfaces because the mutant displays both defective cuticle and exine development^33^.

Maize is one of the most important crops worldwide. Many male sterile mutants have been collected at the stock center of maize MaizeGDB (http://www.maizegdb.org/data_center/phenotype?id=24992), but nevertheless, only four genes involved in pollen exine development have been reported. *MALE STERILE26* (*MS26*) encodes a P450 family protein, which is orthologous to *CYP704B1* in *Arabidopsis* ^34^. *MS45* encodes a strictosidin synthase, which serves as a vital component in seed production technology^35^. *IRREGULAR POLLEN EXINE1* encodes a putative glucose-methanol-choline oxidoreductase^36^. *ABNORMAL POLLEN VACUOLATION1* encodes another P450 family protein that functions in the fatty acid hydroxylation pathway^37^. Here, we report a complete maize male sterile mutant *male sterile 6021* (*ms6021*), which shows defective anther cuticle and exine development. We isolated the monofactorial recessive, nuclear male sterile gene using map-based cloning. The expression pattern analysis showed that *MS6021* was specifically expressed in the tapetum and microspore after meiosis, and MS6021 was mainly localized to the plastid via the N-terminal transit peptide. *MS6021* could functionally complement the *Arabidopsis ms2* mutant, indicating that *MS6021* was the putative maize ortholog of *MS2* and may also function as a fatty acyl-ACP reductase. This work would improve our understanding of anther cuticle and exine development in maize.

## Results

### Phenotypic and genetic analysis of the *ms6021* mutant

**Figure 1:**
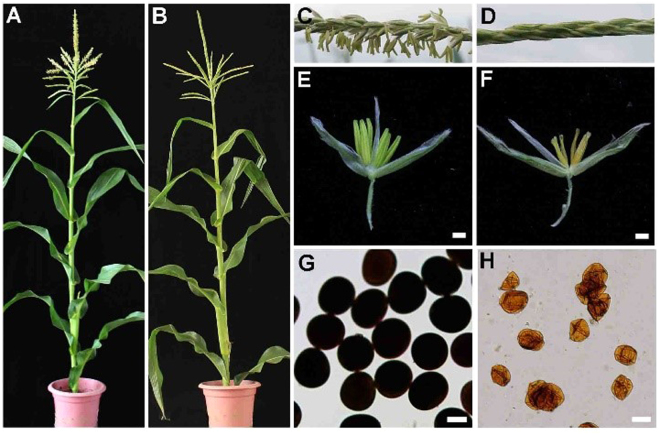
Phenotypic comparison between wild-type and the *ms6021* mutant. (**A**,**B**) Wild-type (**A**) and *ms6021* mutant (**B**) plants at the flowering stage. (**C**,**D**) Branches of wild-type (**C**) and the *ms6021* mutant (**D**) at the flowering stage. (**E**,**F**) Spikelet of wild-type (**E**) and the *ms6021* mutant (**F**) before pollen loss. (**G**,**H**) Pollen grains of wild type (**G**) and the *ms6021* mutant (**H**) stained with a 1% I_2_-KI solution at the flowering stage. Bars = 1 mm in (**E**,**F**) and 50 µm in (**G**,**H**).

To identify maize genes that contribute to anther development, we requested a series of male sterile mutants from the stock at maizeGDB. Among these materials, *ms6021* displayed normal female development but smaller anthers before flowering (Fig. 1D and F) and complete male sterility compared with the wild type (Fig. 1A,C and E). The phenotype was identical to the phenotypic description from MaizeGDB. It was first reported by Patterson E. B. in 1995. I_2_-KI pollen staining revealed an absence of mature pollen in *ms6021* (Fig. 1H) compared with wild-type anthers (Fig. 1G).

When the *ms6021* plants were pollinated with wild-type (B73) pollen, all the F_1_ progeny displayed normal male fertility, indicating that *ms6021* was a recessive mutant. The BC_1_F_1_ population was developed by crossing *ms6021* mutant plants with the F_1_ plants. BC_1_F_1_ fertility testing showed a segregation of 76 normal and 79 mutant plants (χ^2^ = 0.03, P > 0.05), indicating a monofactorial recessive characteristic of *ms6021*. According to the information provied by maizeGDB, *ms6022* (928P), *ms6046* (928S) and *ms6047* (928T) are allelic to *ms6021* (928O). Our allelic testing confirmed the allelic relationship (Supplementary Table S1).

### Defects of the *ms6021* anther development

**Figure 2:**
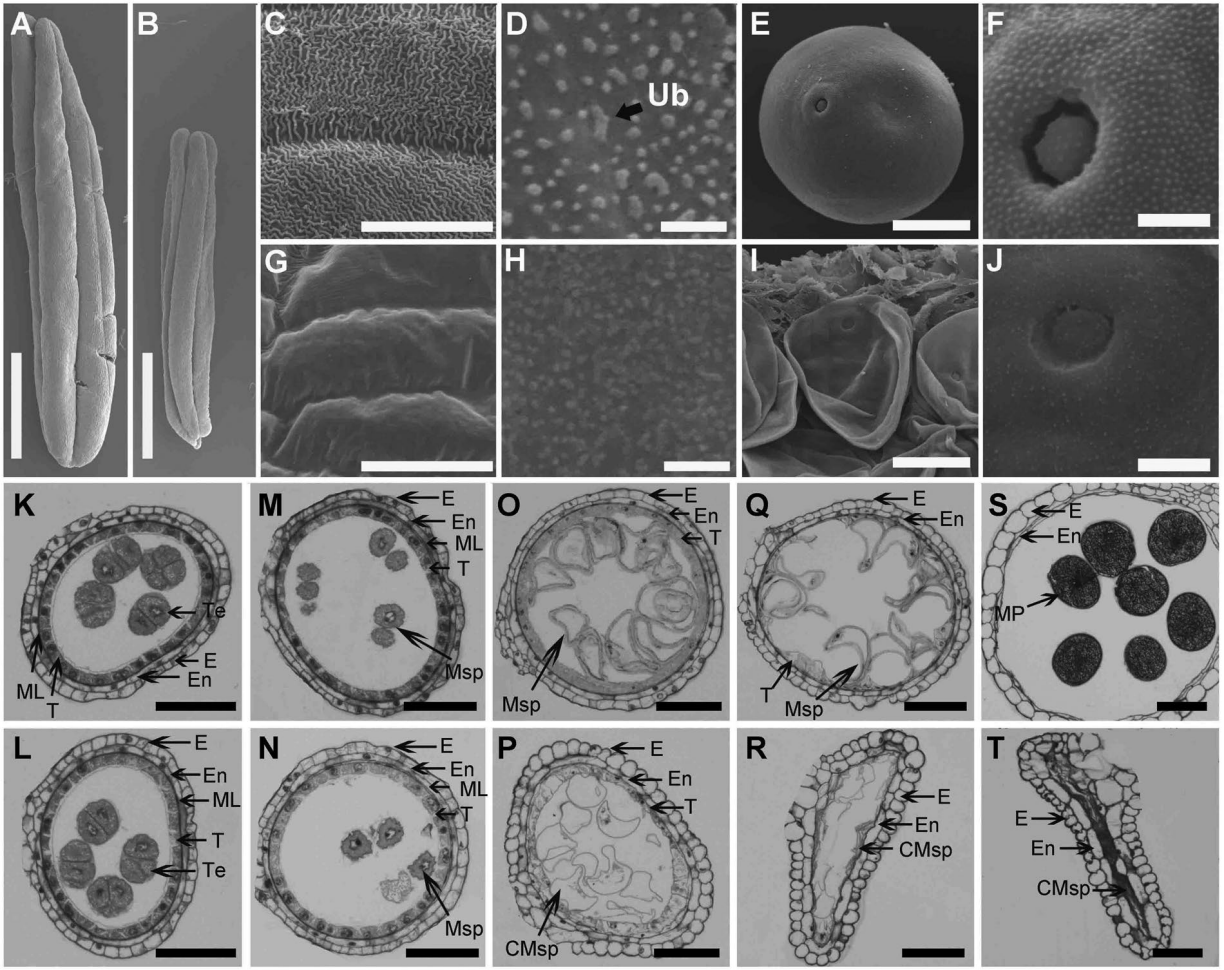
Defective development of the *ms6021* anther surface and pollen wall. (**A**,**B**) Anthers of wild-type (**A**) and *ms6021* (**B**) at the mature pollen stage. (**C**,**G**) SEM analysis of the anther surface of wild-type (**C**) and *ms6021* (**G**) at the mature pollen stage. (**D**,**H**) SEM analysis of the inner surface of wild-type (**D**) and *ms6021* (**H**) at the mature pollen stage. (**E**,**F**,**I**,**J**) SEM analysis of the pollen grain (**E**,**I**) and pollen surface (**F**,**J**) of wild type (**E**,**F**) and (**I**,**J**) at the mature pollen stage. (**K**) to (**T**) Cytological comparison of anther development in wild type and *ms6021* at different stages. The anthers of wild type are shown in (**K**,**M**,**O**,**Q** and **S**); and of the *ms6021* mutant are shown in (**L**,**N**,**P**,**R** and **T**) tetrad stage (**K**,**L**); uninucleate stage (**M**,**N**); large vacuole stage (**O**,**P**); binucleate stage (**Q**,**R**); mature pollen stage (**S**,**T**). CMsp, collapsed microspore; E, epidermis; En, endothecium; ML, middle layer; MP, mature pollen; Msp, microspore; T, tapetum; Te, tetrad; Ub, ubisch body. Bars = 1 mm in (**A**,**B**), 20 µm in (**C**) to (**E**) and (**G**) to (**I**), 5 µm in (**F**,**J**), and 50 µm in (**K**) to (**T**).

To investigate the detailed differences between the *ms6021* mutant and wild type, we used scanning electron microscopy to examine the anther and pollen surfaces at different anther development stages. The anthers of *ms6021* (Fig. 2B) were much smaller than those of wild-type (Fig. 2A) at the mature pollen stage. A three-dimensional reticular structure formed on wild-type epidermal cells (Fig. 2C), while the anther surface of *ms6021* was glossy and smooth (Fig. 2G). At the uninuclear stage of wild type, a large number of granular Ubisch bodies were secreted from the tapetum on the inner locule surface (Fig. 2D). These sporopollenin precursor carriers would be accumulated outside of microspores to form a particulate exine pattern (Fig. 2F), and this process was critical for mature pollen formation (Fig. 2E). At the same stage, much smaller spot-like Ubisch bodies were observed on the inner side of the *ms6021* tapetum (Fig. 2H), indicating unregular aliphatic component transportation to the microspore surface of the mutant. The pollen granule shrunk severely and adhered to the inside of the *ms6021* anther wall (Fig. 2I), and its pollen surface was also smoother than that of wild type (Fig. 2J).

Next, we performed a morphological analysis to identify anther developmental defects in the *ms6021* mutant. Light microscopy was used to examine transverse sections of wild-type and *ms6021* anthers. The tetrads formed normally in the locules of both wild type (Fig. 2K) and the *ms6021* mutant (Fig. 2L). The callus surrounding the tetrads was then digested, and the microspores were easily released (Fig. 2M and N) as previously reported^38^. During the large vacuole stage, exine assembly was completed. The microspores rapidly inflated. Tapetal cells were squeezed, and the middle layer was almost invisible (Fig. 2O). By contrast, in the *ms6021* mutant anther, a tenuous exine was formed around the irregular microspores. The tapetal cells were swollen, and the cellular outline of middle layer were still visible (Fig. 2P). The microspores then entered the trinucleate stage through two cycles of mitotic divisions in wild type. The tapetum nearly disappeared at this stage (Fig. 2Q). Mature pollen grains were formed immediately before flowering in the wild-type anther (Fig. 2S). In contrast, the *ms6021* anther started to shrink (Fig. 2R) and developed into a rectangular structure (Fig. 2T).

**Figure 3:**
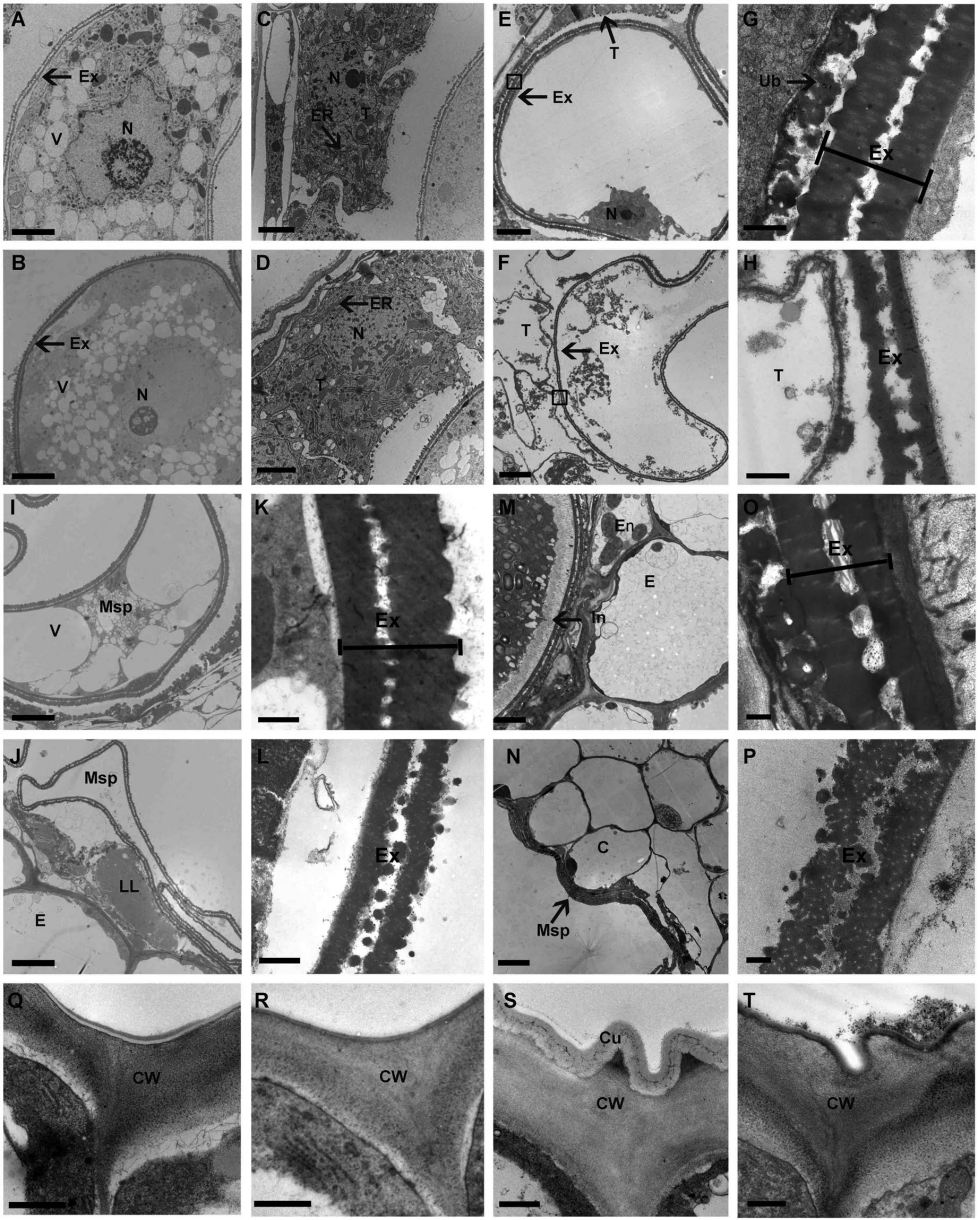
TEM analysis of anthers from wild-type and *ms6021*. (**A**) to (**D**) Microspore (**A**,**B**) and tapetum (**C**,**D**) of anthers from wild type (**A**,**C**) and the mutant (**B**,**D**) during the uninucleate stage. (**E**) to (**H**) Microspore (**E**,**F**) and pollen exine (**G**,**H**) of anther from wild-type (**E**,**G**) and mutant (**F**,**H**) at large vacuole stage. (**I**) to (**L**) Microspore (**I**,**J)** and pollen exine (**K**,**L**) of anthers from wild type (**I**,**K**) and the mutant (**J**,**L**) at the binucleate stage. (**M**) to (**P**) Anther wall (**M**,**N**) and pollen exine (**O**,**P**) of anthers from wild type (**M**,**O**) and the mutant (**N**,**P**) at the mature pollen stage. (**Q**) to (**T**) Anther epidermal surface of wild type (**Q**,**S**) and the *ms6021* mutant (**R**,**T**) at the uninucleate stage (**Q**,**R**) and mature pollen stage (**S**,**T**). C, cavity for dehiscence. Cu, cuticle; CW, cell wall; ER, endoplasmic reticulum; Ex, exine; In, intine; LL, lipidosome-like; Msp, microspore; N, nucleus; T tapetum; Ub, ubisch body; V, vacuole. G and H were zoomed form black solid boxes region in E and F respectively. Bars = 5 µm in (**A**,**B**,**E**,**F**,**I**,**J**,**M** and **N**), 2 µm in (**C**,**D**), and 0.5 µm in (**G**,**H**,**K**,**L**,**O** and **P**) and (**Q**) to (**T**).

To gain more detailed insight into the defective anther development of *ms6021*, transmission electron microscopy (TEM) was performed. In accordance with the light microscopy results, the abnormality did not appear until the uninucleate stage. In both wild-type and *ms6021* anthers, multiple small vacuoles were observed in the microspores, and the structural primexine of the microspores was clearly observed (Fig. 3A and B). The heavily stained tapetal cells revealed a vigorous metabolism (Fig. 3C and D). Subsequently, the small vacuoles merged into a central large vacuole, and the tapetum was squeezed together to form a hill-like structure (Fig. 3E) in the wild-type anther. Moreover, a large amount of Ubisch bodies were secreted out of the tapetum, which transported the sporopollenin precursor to the outside surface of the microspore. The exine rapidly thickened (Fig. 3G). By contrast, both the microspore and tapetum collapsed and the cell contour was remained in the *ms6021* anther (Fig. 3F) at the vacuolated stage. In addition, we did not observe any normal Ubisch bodies in the *ms6021* locule, and the exine was much thinner than that in wild type (Fig. 3H). The microspore underwent one round of mitosis and entered the binucleate stage in wild type. The large vacuole was divided, and the microspore shrunk. Programmed cell death (PCD) was launched in the tapetum (Fig. 3I) and exine morphogenesis ended (Fig. 3K). By comparison, the microspore contents of *ms6021* completely disappeared. Abnormal liposome-like structures were formed in the tapetal cell (Fig. 3J). Normal exine thickening did not occur in the mutant anther (Fig. 3L). During the trinucleate stage of wild type, the tapetum and middle layer were invisible, and only the endothecium and epidermis remained in the anther wall. The intine, which was composed of polysaccharides, was accumulated inside the exine (Fig. 3M and O). The tapetum did not disappear completely in the *ms6021* anther compared with wild type. The microspores further shrunk and adhered tightly to the anther wall (Fig. 3N). The intine did not form in the mutant (Fig. 3P). We also studied the anther cuticle development process. At the uninucleate stage, there was no cuticle structure outside of the epidermal cells of either wild type or the *ms6021* mutant (Fig. 3Q and R). Before flowering, a hair-like cuticle formed on the surface of the wild-type epidermis (Fig. 3S). The outmost layer of *ms6021* remained glossy (Fig. 3T), which was consistent with the SEM results (Fig. 2D). An identical cytological analysis was performed using the *ms6047* mutant. Interestingly, the anther developmental process in this mutant was more completely disrupted compared with the *ms6021* mutant (Supplementary Figure S1).

### Aliphatic alteration of *ms6021* anther

**Figure 4:**
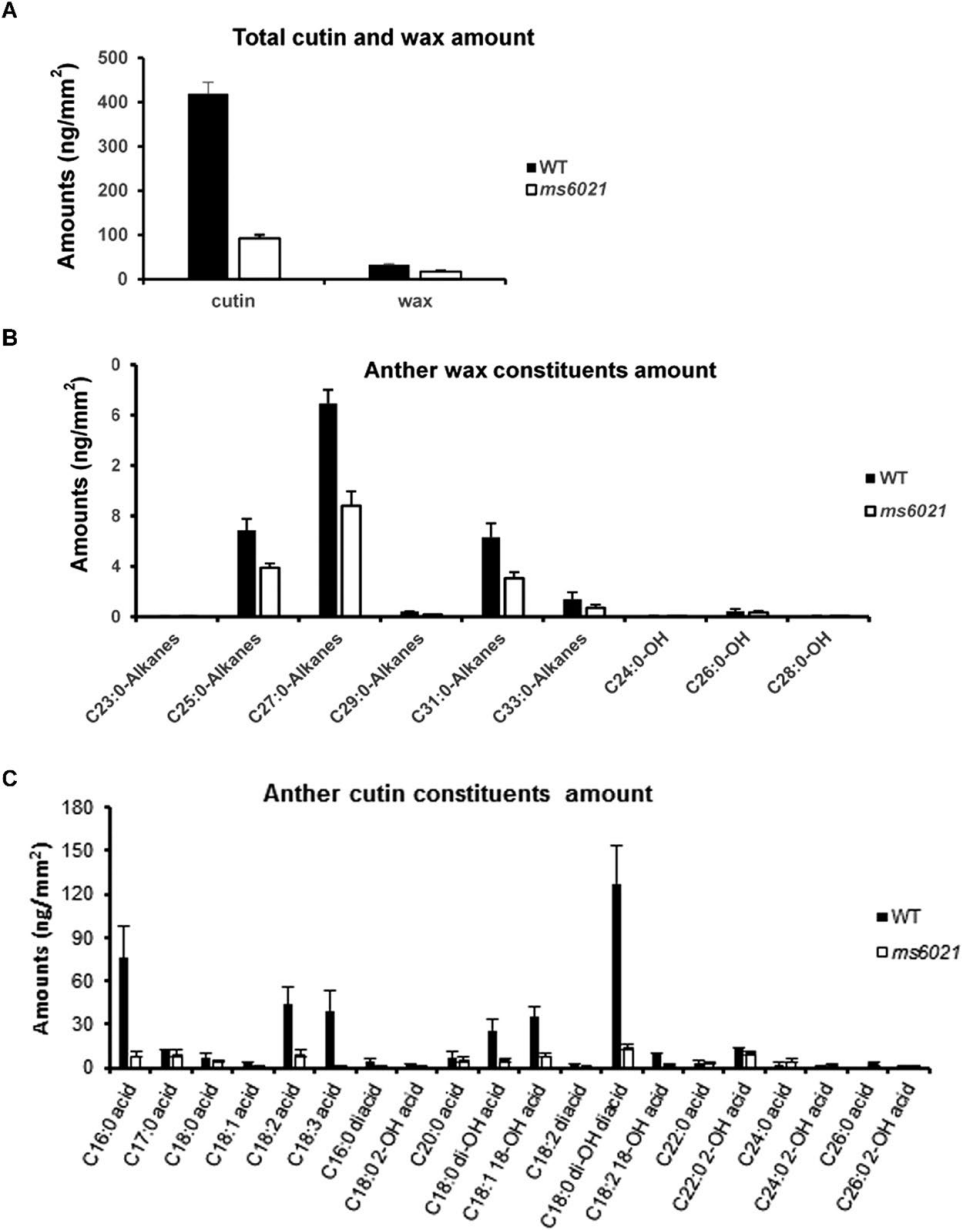
Analysis of anther wax and cutin monomers in wild type and the *ms6021* mutant. (**A**) Total cutin and wax amounts per unit area (ng/mm^2^) in wild-type (black bars) and *ms6021* (white bars) anthers. Error bars indicate the SD (n = 3). (**B**) Wax amounts per unit (ng/mm^2^) in wild type (black bars) and *ms6021* (white bars) anthers. Error bars indicate the SD (n = 3). (**C**) Cutin amounts per unit (ng/mm^2^) in wild-type (black bars) and *ms6021* (white bars) anthers. Error bars indicate the SD (n = 3).

The smooth anther surface, abnormal Ubisch bodies, and defective pollen exine structure indicated a disruption of the accumulation of aliphatic components in the *ms6021* anther. Next, we extracted cuticle wax, cutin and soluble fatty acid (SFA) from wild-type and *ms6021* anthers step-by-step, and analyzed the composition by GC-MS^39,40^. The methods described by Li^7^ was used to plot surface area against fresh weight of corresponding samples (Supplementary Figure S2). The total wax was 32.58 ng/mm^2^ and 17.19 ng/mm^2^ in wild-type and *ms6021* anthers, respectively, representing a 47.23% decrease in the mutant anther (Fig. 4A). C23, C25, C27, C29, C31, and C33 alkenes and C24 and C26 alcohols were significantly decreased in the mutant (P < 0.01) (Fig. 4B; Supplementary Table S2). The total amount of cutin in *ms6021* anthers (92.93 ng/mm^2^) decreased by 78% compared with that of wild type (419.48 ng/mm^2^, P < 0.01; Fig. 4A). All monomer compositions less than 24 carbons were significantly reduced in the mutant anthers (Fig. 4C; Supplementary Table S3). The amount of total soluble fatty acids with carbon lengths ranging from 16 to 28 was 20.05 µg/mg in wild-type anthers. By contrast, in the mutant anthers, the total amount of soluble fatty acids was reduced to 2.81 µg/mg (Table 1).

**Table 1:** Total soluble fatty acids of wild-type and ms6021 anthers.

Soluble fatty acids	Wide Type *ms6021*		Down
	Mean ± SD (ng/mg dry weight)		
C16:1 acid	0.001 ± 0.0004	0	100%
C16:0 acid	8.838 ± 1.759	0.479 ± 0.086	94.58%
C18:2 acid	4.548 ± 0.551	0.596 ± 0.114	86.90%
C18:3 acid	5.241 ± 1.229	0.082 ± 0.015	98.43%
C18:1 acid	0.458 ± 0.042	0	100%
C18:0 acid	0.312 ± 0.036	0.283 ± 0.039	9.11%
C20:0 acid	0.217 ± 0.019	0.381 ± 0.035	−75.25%
C22:0 acid	0.057 ± 0.010	0.158 ± 0.018	−175.43%
C24:0 acid	0.056 ± 0.008	0.227 ± 0.046	−307.69%
C26:0 acid	0.021 ± 0.003	0.113 ± 0.028	−431.06%
C28:0 acid	0.102 ± 0.014	0.301 ± 0.129	−193.75%
Total	20.055 ± 3.404	2.804 ± 0.417	86.03%

### Isolation of *MS6021*

**Figure 5:**
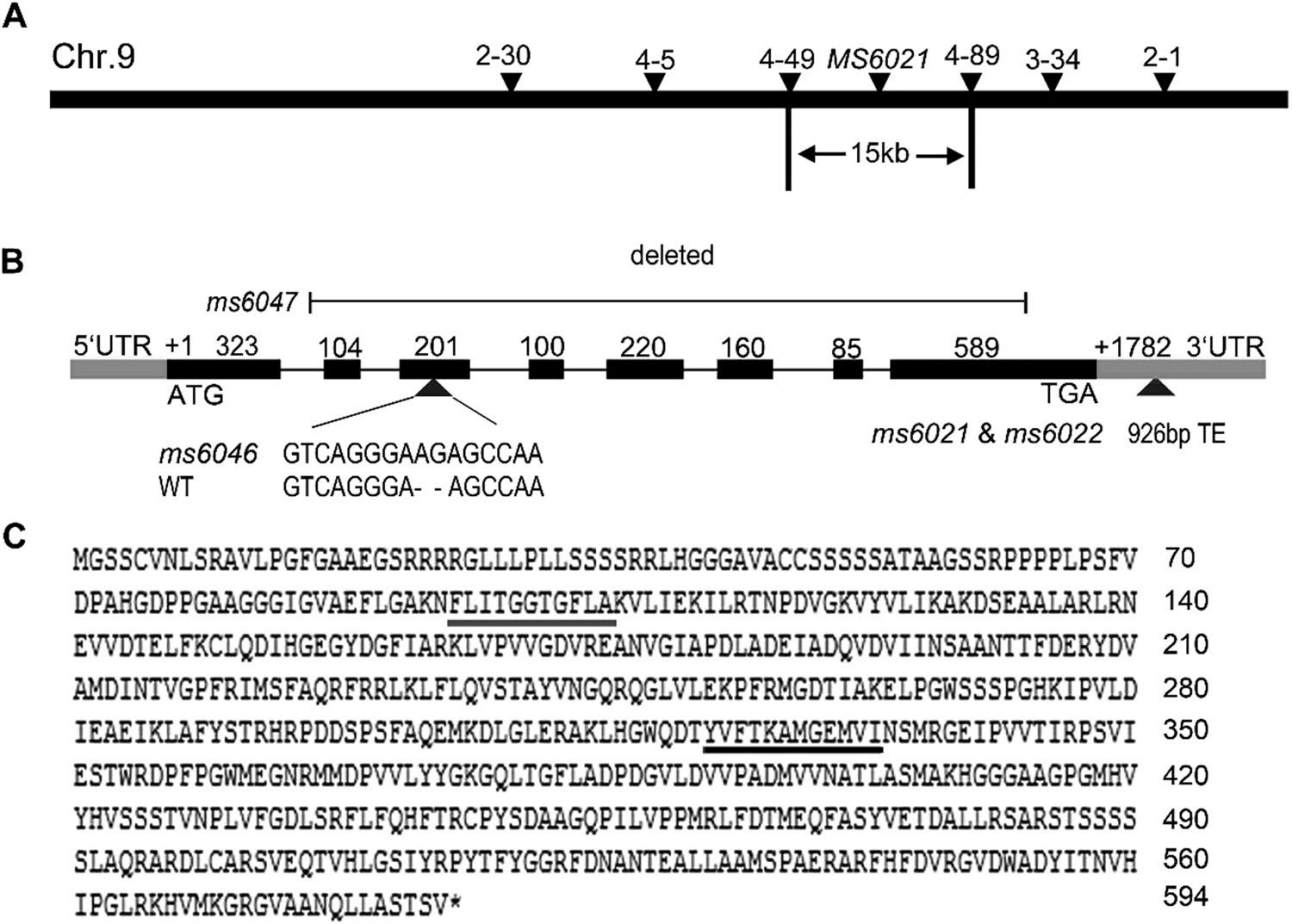
Molecular cloning and sequence analysis of *MS6021*. (**A**) Fine mapping of the *MS6021* on chromosome 9. The location and name of the makers are indicated. The MS6021 locus was mapped to a 15-kb region between markers 4–49 and 4–89. (**B**) A schematic representation of the exon and intron structure of *MS6021*. The black boxes indicate exons, grey boxes indicate UTR region, and intervening lines indicate introns. +1 indicates the start codon (ATG); +1782 indicates the stop codon (TAA). A 926-bp fragment is inserted into the 3′UTR in *ms6021* and *ms6022*; 2-bp is inserted into the third exon in *ms6046*; a 2142-bp fragment from the first intron to the last exon is deleted in *ms6047*. (**C**) The amino acid sequence of MS6021. The grey and black lines underlining indicated putative NAD-binding region and active region respectively.

Map-based cloning was used to isolate *MS6021*. The gene was initially mapped to a 5.93-Mb interval on chromosome 9 between markers 2–30 and 2-1 (Fig. 5A). Then 998 individuals from the BC_1_F_1_ population were used for fine mapping. *MS6021* was mapped to a 15-kb region based on the B73 reference genome. There were two complete open reading frames in this region (*GRMZM2G420926 & GRMZM2G120987*). The *GRMZM2G420926* genomic sequence was identical between wild type and *ms6021*. There was a 926-bp insertion in the 3′ untranslated region (UTR) of *GRMZM2G120987* in the *ms6021* genome. The *GRMZM2G120987* genomic sequences of the three other alleles were then analyzed. The sequencing results revealed that the genomic sequence of *ms6022* was identical to that of *ms6021*, two residues were inserted into the third exon in *ms6046* resulting in a frame shift, and a 2105-bp region from the first intron to the last exon was deleted in *ms6047* (Fig. 5B). The above results indicate that variations of the *GRMZM2G120987* sequence are responsible for the phenotypic defects in the mutant anther. The transcribed region of *GRMZM2G120987* includes a 279-bp 5′ UTR, a 482-bp 3′ UTR, and a 1782-bp ORF encoding 593 amino acids (Fig. 5B).

### *MS6021* is mainly expressed in the tapetum and microspore

**Figure 6:**
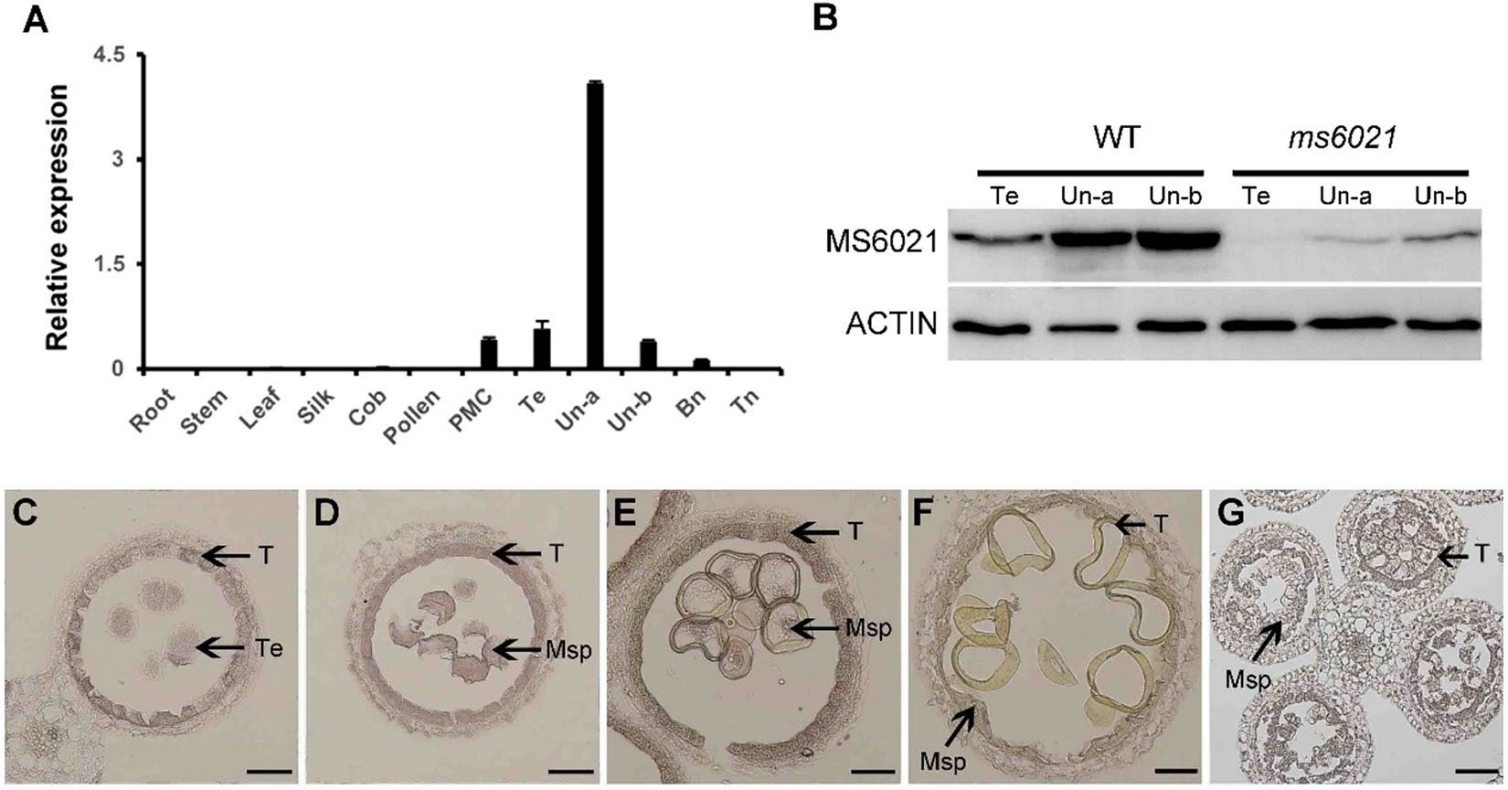
Expression pattern of *MS6021*. (**A**) qRT- PCR analysis of *MS6021* expression. Error bars indicate the SD (n = 3). (**B**) Protein gel blot analysis. Full-length gels are presented in Supplementary Figure S4. Te, tetrad stage; Un-a, early uninucleate stage; Un-b, late uninucleate stage. (**C**) to (**F**) RNA *in situ* hybridization using an anti-sense probe during the tetrad stage (**C**), early uninucleate stage (**D**), large vacuole stage (**E**), and binucleate stage (**F**). (**G**) RNA *in situ* hybridization using a sense probe during the early uninucleate stage. Msp, microspore; T, tapetum. Bars = 50 µm in **(C**) to (**F**) and 100 µm in (**G**).

To understand the expression pattern of *MS6021*, qRT-PCR analysis was performed using total RNA isolated from both vegetative and reproductive organs. The results indicated that *MS6021* was not expressed in vegetative and female reproductive organs. Only trace amount of *MS6021* was detected in the pollen mother cell (PMC) stage and tetrad stage, peaking during the early nucleate stage and then rapidly declining (Fig. 6A). Its expression level significantly decreased in the mutant during the uninucleate stage (Supplementary Figure S3). *In situ* hybridization was performed to confirm the spatial and temporal expression pattern of *MS6021* in wild-type anther sections. The *MS6021* transcript could be detected in both microspores and tapetum from the tetrad to the binucleate stage (Fig. 6C–F); high-level expression was detected at uninucleate stage (Fig. 6D), while only background signal was detected using the sense probe during the same stage (Fig. 6G), which was consistent with the qRT-PCR results. Moreover, we produced polyclonal antibodies to perform the western blot analysis. Trace amounts of MS6021 were detected in the anthers of the *ms6021* mutant (Fig. 6B), which may underlie the phenotypic differences between *ms6021* and *ms6047* (Fig. 2 and Supplementary Figure S1).

### MS6021 is localized to plastids

**Figure 7:**
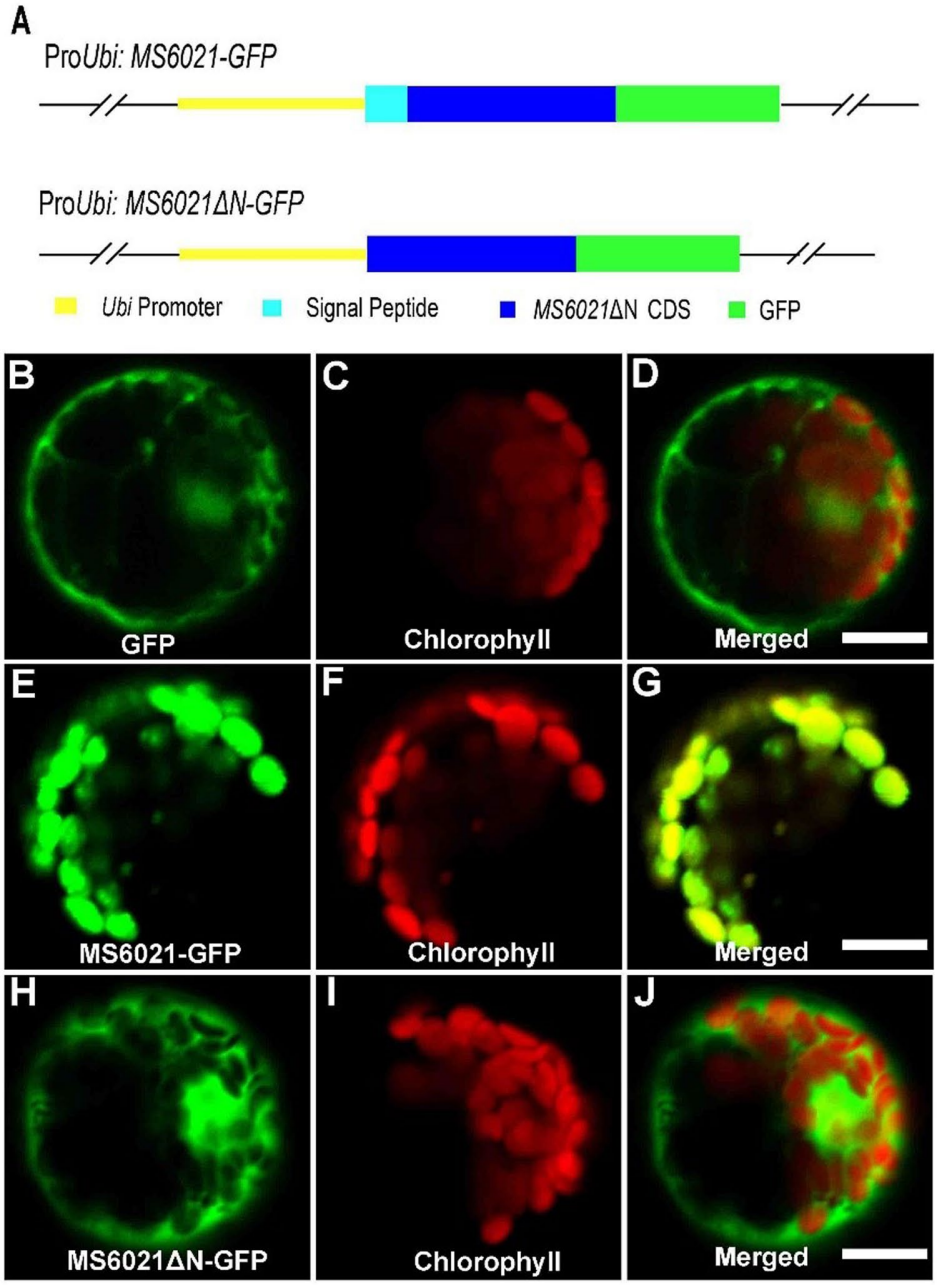
Subcellular localization analysis of MS6021. (**A**) Diagram of the full-length constructs of *MS6021* cDNA and signal region deleted cDNA fused to GFP under the control of the maize ubiquitin promoter. (**B**) to (**D**) A maize protoplast expressing empty pJIT163-GFP showing green fluorescence (**B**), chlorophyll autofluorescence (**C**), and the merged signals (**D)** of (**B**) and (**C**). (**E**) to (**G**) A maize protoplast expressing fused MS6021-GFP showing green fluorescence (**E**), chlorophyll autofluorescence (**F**), and the merged signals (**G**) of (**B**) and (**C**). (**H**) to (**J**) A maize protoplast expressing empty fused MS6021ΔN-GFP showing green fluorescence (**H**), chlorophyll autofluorescence (**I**), and the merged signals (**J)** of (**H**) and (**I**). Bars = 10 µm.

The targetP 1.1 server (http://www.cbs.dtu.dk/services/TargetP/) was used to analyze the amino acid sequence of MS6021. There was a chloroplast signal peptide predicted at the N-terminal of MS6021. To identify the subcellular localization of MS6021, we constructed plasmids containing *MS6021-GFP*, *MS6021*Δ*N-GFP* (the predicted signal peptide was deleted), or *GFP* only driven by the maize ubiquitin promoter **(**Fig. 7A). These plasmids were then introduced into protoplasts isolated from young maize leaves. The plastid signal was detected by chlorophyll autofluorescence. The MS6021-GFP signal co-localized with the autofluorescence of chlorophyll in the plastid (Fig. 7E–G). In contrast, the GFP alone signal (Fig. 7B–D) and the MS6021ΔN-GFP (Fig. 7H–J) signal were observed in the cytoplasm and did not co-localize with the autofluorescence of chlorophyll. This result revealed that MS6021 was localized in plastids mediated by N-terminal signal peptide.

### Functional conservation of *MS6021*

**Figure 8:**
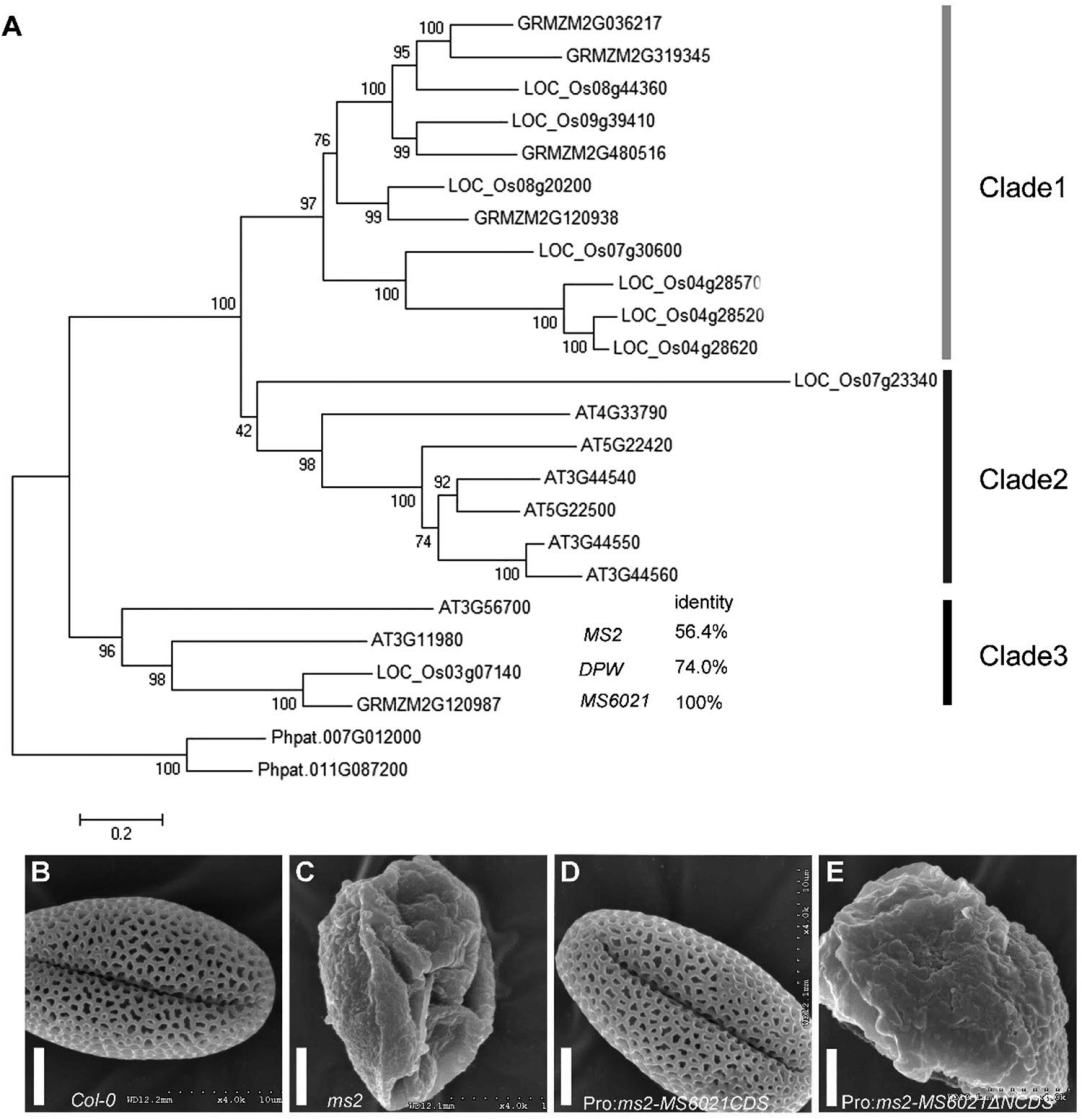
Functional conservation of *MS6021*. (**A**) A neighbor-joining phylogenetic tree summarizing the evolutionary relationships among FAR members in *Arabidopsis*, rice, and maize of (BLASTPE E-value < 1E-100). The proteins are named according to their Phytozome accession numbers. The numbers under the branches refer to the bootstrap value of the neighbor-joining phylogenetic tree. The length of the branches is proportional to the amino acid variation rates. At, *Arabidopsis thaliana*; Os, *Oryza sativa*; Zm, *Zea mays*. The bar indicates the estimated number of amino acid substitutions per site (for the protein alignment, see Figure S4). (**B**) to (**E**) SEM analysis of the pollen wall surface of wild-type (**B**), *ms2* mutant (**C**), and transgenic *ms2* mutant lines (**D** and **E**) at anthesis. Bars = 5 µm.

According to the phytozome11 information, MS6021 contained a NAD-binding domain and a sterile domain (Fig. 5C), and it is considered to be a member of the fatty acyl-CoA reductase family (FAR). The FAR is a cluster of reductases that catalyze the transformation of fatty acyl-CoA/ACP to the corresponding alcohol and comprises 5, 9 and 8 members in maize, rice and *Arabidopsis*, respectively. We compared the amino acid sequences of these 22 homologous genes (Supplementary Figure S5). A neighbor-jointing (NJ) tree was subsequently constructed with orthologous genes from *Physcomitrella patens* as an outgroup (Fig. 8A). The NJ tree was grouped into three clades. Clade 1 was specific to monocot, and all the genes in this clade were from maize or rice. In clade 2, all the genes were from *Arabidopsis* except *LOC_Os07g23340* from rice. Clade 3 was composed of *AT3G56700* and three male sterile genes, namely *MS2*, *DPW* and *MS6021*. *AT3G56700* was also a plastid-localized protein that was specifically expressed in anthers. It may be functionally redundant with MS2, and it is responsible for partial fertility in the *Arabidopsis ms2* mutant^16,41^.

The phylogenetic analysis indicated that *MS6021*, *MS2* from *Arabidopsis*, and *DPW* from rice are orthologs, and *ms2* ^18^ and *dpw* ^19^ mutants display a similar phenotype to *ms6021*. However, MS6021 only shares 56.4% identity with MS2 (Supplementary Figure S5). To identify the evolutionary relatedness of these two genes, a complementation experiment was performed with the *MS6021* complete CDS region and the CDS region lacking the signal peptide driven by the *MS2* native promoter. The two constructs were used to transform the *Arabidopsis ms2* mutant. Five positive lines per transformation were selected to assess the pollen morphology by SEM. The results showed that the CDS of *MS6021* was able to rescue the phenotype of *ms2*, whereas the CDS lacking the signal peptide failed to rescue the *ms2* mutant (Fig. 8B–E). These results revealed the functional conservation of MS2/MS6021 between *Arabidopsis* and maize.

### The *ms6021* mutant exhibited abnormal expression of genes involved in aliphatic metabolism

To better understand the defects in *ms6021*, RNA-seq was performed with total RNA isolated from uninucleate-stage anthers of wild type and *ms6021* using three biological repeats. High-throughput sequencing was performed with the Illumina Hiseq2500 platform. More than 30 million reads were generated for each sample. After filtration of the data, the clean reads were aligned to the maize genome references^42^. There were 23273 genes transcripts detected in wild type and *ms6021* mutant. We identified 1163 differentially expressed genes (DEGs) with a threshold fold change greater than 2 and a false discovery rate less than 0.05. Among the DEGs, 594 genes were up-regulated and 569 genes were down-regulated (Supplementary Figure S6A). The RNA-seq results were confirmed by qRT-PCR (Supplementary Figure S7). The GO analysis revealed that multiple processes related to pollen maturation were impacted in *ms6021* (Supplementary Figure S6B), including pollen wall assembly (GO:0010208), NADP metabolic processes (GO:0006739), oligosaccharide metabolic processes (GO:0009311), secondary metabolic processes (GO:0019748) and extracellular matrix organization (GO:0030198). Plant immune system-related processes, such as the response to stress (GO:0006950) and positive regulation of defense responses (GO:0031349), were also simulated in *ms6021*.

It is well known that sporopollenin is composed of fatty acids and phenolic^43^. The precise components of phenolic are largely unknown. They are the main products of phenylalanine metabolism, including flavonoids, stilbenes, coumarins and lignin^25^. The KEGG pathway analysis indicated that both the metabolisms of fatty acids and phenolic were affected in *ms6021* (Supplementary Table S4). More genes involved in phenylalanine biosynthesis and flavonoid biosynthesis showed altered expression patterns in comparison to the genes involved in fatty acid metabolism, such as fatty acid elongation, fatty acid biosynthesis and fatty acid unsaturation in the mutant. In addition, the expression of ABC transporter proteins, which function as sporopollonin precursor transporters, was also affected. Taken together, these findings indicated the severely disrupted metabolism of fatty acid-derived components in the *ms6021* anthers.

## Discussion

Both the cuticle and pollen wall have a strategic position at the interface between the plant and environment. They must protect genetic material from various stresses. Deciphering the chemical composition of these protective tissues has encountered enormous challenges due to their extreme resistance to degradation and sophisticated fine structures. Recently, several male sterile mutants associated with defective anther cuticle or/and pollen wall development have been identified using genetic approaches, including *acos5* ^24^, *cyp703a2* ^21^, *cyp704b1* ^22^, *drl1* ^27^, *lap5*/*6* ^25^, *ms2* ^18^, in *Arabidopsis*, and *cyp703a3* ^20^, *cyp704b2* ^7^, *dpw* ^19^, *osabcg15* ^44^, *osabcg26* ^45^, *tdr* ^6^, *wda1* ^10^ in rice. In addition, some reviews have summarized the regulation network underlying cuticle and pollen wall development of these model plants^4,8,23^. However, only four genes, *MS26* ^34^, *MS45*, *IPE1* ^36^, and *APV1* ^37^, have been reported to participate in cuticle or/and pollen wall development in maize. All the four cloned genes encoded enzymes involved in aliphatic metabolism, while their exact biochemical function and substrate relationships are largely unknown.

We reported here the male sterile mutant *ms6021* in maize, which also displayed defective cuticle and pollen wall development. The epidermal anther surface of *ms6021* was smooth and glossy (Fig. 2G), and the TEM results showed that the cuticle layer had largely disappeared (Fig. 3T). The total amount of wax and cutin decreased by 47% and 78%, respectively (Fig. 4A; Supplementary Tables S2 and S3). The wax compositions of the mutant showed a moderate (<50%) decrease in both fatty alcohols and alkanes (Supplementary Table S2). By contrast, most of the cutin compositions decreased by more than 75% in the mutant anthers (Supplementary Table S3). This result indicated that the products of MS6021 most likely served as dominant precursors for cutin biosynthesis, not the dominant substrates for fatty acid elongation pathway. The conclusion was in accordance with KEGG analysis result that few DEGs were enriched in fatty acid elongation pathway. Metabolism analysis also showed that the proportion of total soluble fatty acids decreased to 13.97% (Table 1). The detailed constituent variation revealed a drastic reduction (up to 100%) in C16 - C18 fatty acids and a sharp increase (2-5-fold) in VLCFAs (>C20; Table 1). The results were consistent with the putative palmitic acyl ACP reductase function of MS6021 and further confirmed that the product of MS6021 was not the dominant substrate for VLCFA biosynthesis.

Aborted pollen grains were observed in the anther locule of *ms6021* (Fig. 2I). Ubisch bodies, sporopollenin trafficking vehicles between the tapetum and pollen wall, disappeared from the inner surface of the mutant (Figs. 2H and 3H). Insufficient material supplementation led to failed exine thickening in *ms6021* (Fig. 3L and P) after microspores release from the tetrads. Based on chemical analysis, it was assumed that sporopollenin was composed of aliphatic derivatives and a mixture of phenolic^43,46,47^. Phenolic is a large class of secondary metabolites^48^, which are mainly the products of phenylpropanoid metabolism. Flavonoids belong to a subclass of phenolic compounds. They are important for plant fertility in maize^49^, petunia^50^, and tobacco^51^. Among the *Arabidopsis* genes required for sporopollenin formation, *ACYL COENME A SYNTHETASE5* ^24^, *LAP5*/*6* ^25,26^, and *TETRAKETIDE α-PYRONE REDUCTASE* ^27,28^ function in the synthesis of hydroxylated tetraketide α-pyrones. *TRANSPARENT TESTA4* ^52^ and *4-COUMARATE:COENZYME A LIGASE* ^53^ encode enzymes that participate in flavonoid biosynthesis. Both polyketides and flavonoids are believed the major composition of sporopollenin^28^. KEGG analysis revealed that DEGs were enriched in phenylpropanoid biosynthesis, phenylalanine metabolism, and flavonoid-related derivative biosynthesis and metabolic pathways (Supplementary Table S4). The orthologs of the above-mentioned genes (*GRMZM2G108894*/*LAP5*, *GRMZM2G380650*/*LAP6*, *GRMZM2G422750*/*TT4*, *GRMZM2G004683*/*TKPR1*) were also included in these pathways. The expression of *IPE1* ^36^, a newly reported male sterile maize gene that participated in cutin and wax biosynthesis, was influenced in *ms6021* anthers as well. The expression of genes involved in maize anther cuticle and sporopollenin biosynthesis in *apv1*, *ipe1* and *ms6021* were compared. Most genes displayed different expression change (Supplementary Table S5), which was in accordance with differences of lipidomic alteration.

In *Arabidopsis* and rice, the ATP-binding cassette transporter has been reported to be required for sporopollenin accumulation. It has been proposed to be responsible for sporopollenin trafficking out of the tapetum^29,30,32,44,54^. The altered expression of multiple predicted ABC transporter genes (Supplementary Table S4) indicated abnormal sporopollenin trafficking in the mutant. Although genetic approaches provide insights into sporopollenin biosynthesis and composition, the trafficking mechanism of sporopollenin from the tapetum to the exine remains poorly understood^8,55^.

The FAR activity among these orthologous genes of *MS6021* was conserved between monocot and dicot plants for both *DPW* ^19^ and *MS6021* could functionally complement *ms2* of Arabidopsis (Fig. 8D). Nevertheless, their products may not participate in the identical metabolism pathway, because mutants in different species displayed subtle differences in male sterility. The pollen of *ms2* in Arabidopsis presented partial sterility, while both *dpw* in rice and *ms6021* in maize showed complete male sterility. Furthermore, the total wax content of *ms6021* was observably declined, unlike that of *dpw* ^19^ (Fig. 4A).

**Figure 9:**
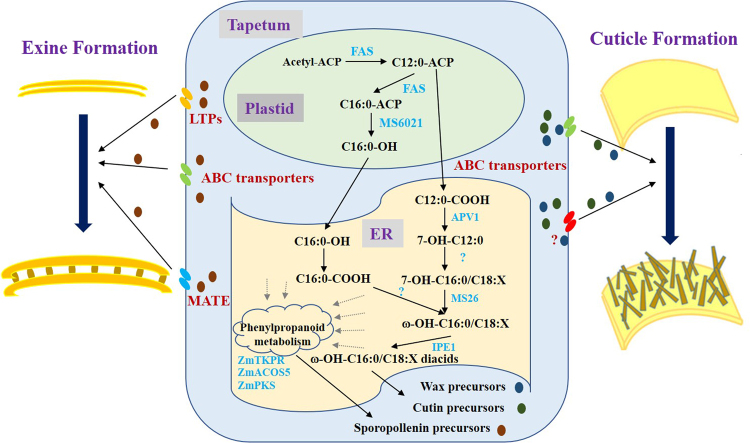
The proposed model of MS6021 function during anther cuticle and exine development in maize. Fatty acids are de novo synthesized in the form of esterified ACP in plastids. The palmitoyl-ACP could be reduced to corresponding alcohol and then be transported from plastids to ER. Hexadecanol is converted to fatty acid^19^. The hydroxylated fatty acid would be oxygenated by P450 to form hydroxy fatty acid, which is further oxygenated by IPE1^43^, or participate in VLFAs biosynthesis. The intermediate metabolites interplayed with phenylpropanoid metabolism, which functioned as supplier for sporopollenin synthesis^8^. Finally, the cutin, wax and sporopollenin precursors are translocated from the tapetal cells into the locule and anther epidermis by ATP binding cassette transporters^29,30,32,44,54^, lipid transport proteins^33^ and multidrug and toxic efflux transporters^4^.

In conclusion, we proposed a working model of how MS6021 participated in maize anther cuticle and exine development (Fig. 9). MS6021 functioned as a fatty acid reductase in plastids in maize. It reduced the palmitoyl-ACP to corresponding alcohol, which would be transferred to the endoplasmic reticulum^18,19^. In the main organelle for fatty acid modification, the palmitoyl alcohol would influence phenylpropanoid biosynthesis, flavonoid biosynthesis, fatty acid enlongation and other fatty acid modification pathways directly or indirectly (Supplementary Table S4). These products would finally serve as precursors for cutin, wax and sporopollenin assembly^8,13^. The defect of *MS6021* resulted in altered expression of massive genes and immature maize anther cuticle and pollen wall, while the regulation network underlying this remains unknown. More work needs to be done to decipher the complicated regulation pathway.

## Methods

### Plant materials and growth conditions

*ms6021*, *ms6022*, *ms6046*, and *ms6047* mutant lines were obtained from the Maize Genetics Cooperation Stock Center. *ms6021* was used to generate the BC_1_F_1_ population with B73. All plants were cultivated in the experimental field of the Institute of Genetics and Developmental Biology, Chinese Academy of Sciences (IGDB, CAS) in Beijing and Hainan Province.

### Phenotypic analysis of *ms6021*

For SEM analysis, fresh anthers from both wild type and the mutant at different stages were immersed in FAA solution (50% ethanol, 5% glacial acetic acid, 5% formalin) for 24 h at room temperature for fixation. The samples were then dehydrated in a serial of ethanol gradients (50–100%). After critical-point drying, the anthers were coated with palladium gold and then observed using a scanning electron microscope (HITACHI S-3400N).

For cytological observation, anthers were pricked and fixed in FAA solution overnight. The samples then were dehydrated using a serial of ethanol (50–100%) and embedded in spurr resin. Semi-thin sections were obtained using a Leica UE, stained with 0.05% toluidine blue and observed with an Olympus BX-53 microscope.

For TEM analysis, fresh anthers were vacuum infiltrated and prefixed in 3.5% glutaraldehyde (with 0.1 M phosphate buffer, pH7.4) followed by rinsing with 0.1 M phosphate buffer. The samples then were transferred into 1% osmium tetraoxide and rinsed with 0.1 M phosphate buffer. After fixation, the samples were dehydrated using an ethanol series from 50% to 100% and embedded in spurr resin. Ultra-thin sections were collected with a Leica EM-UC6. After double staining with uranyl acetate and lead citrate, images were obtained with a HITACHI H-7500 transmission electron microscope.

### Aliphatic components analysis

To calculate the surface area, the anthers were considered as cylinders. The anther surface area was then plotted against the corresponding fresh weight^7^. Cuticle wax, cutin and total soluble fatty acid extraction and GC-MS analysis were performed as described previously^19,43^.

### Isolation of *MS6021*

*MS6021* has been reported to located on chromosome 9. For primary mapping, bulk segregation analysis (BSA) was used to identify pleomorphic markers associated to *MS6021*. The locus was first mapped between two simple sequence repeat (SSR) markers, 2–30 and 2-1, on the long arm of chromosome 9, at a position not far from telomere. Then, 998 individuals were used for fine mapping with pairs of primers (4–5, 4–49, 4–89, and 3–34). The primer sequences are listed in Supplementary Table 6.

### RNA extraction and qRT-PCR

Total RNA was isolated from root, stem, leaf, silk, cob, pollen and different stages of anthers using the RNeasy Plant mini Kit (QIAGEN) as described by the manufacturer. The developmental stage of anthers was determined based on the semi-section morphology. One microgram of total RNA was used to synthesis cDNA using RevertAid First Strand CDNA Synthesis Kit (THERMO). qRT-PCR was performed on the Roche LightCycle480 system with SYBR Green Premix (TAKARA). All PCR reactions were conducted using 40 cycles at 98 °C for 10 s, 60 °C for 10 s, and 72 °C for 10 s, in a 20 µl reaction mixture containing 10 pmol of each primer and 2 µl of cDNA as template. All reactions were performed in triplicate, and *ZmActin1* was used as the internal control for normalization. All primers used for qRT-RCR are listed in Supplementary Table 6.

### *In situ* hybridization

Wild-type anthers at different developmental stages were fixed in FAA solution and then dehydrated in a gradient ethanol series (50%, 70%, 85%, 95% and 100%). After embedding in paraffin, 8 µm thick sections were obtained using a Leica microtome. To generate anti-sense and sense probes, a 750-bp cDNA fragment was produced by PCR amplification. RNA *in-situ* hybridization was performed according to a previously described protocol^56^.

### Western blot analysis

Different developmental stages of anthers from wild type and the *ms6021* mutant were grounded into a powder in liquid nitrogen. Total protein was isolated using Plant Protein Extraction Reagent (CW0885M) according to the manufacturer’s protocol (CWBIO) and quantified by the Bio-Rad protein assay. A small synthetic peptide (C-ESTWRDPFPGWMWNGNR-N) was generated to obtain the polyclonal antibody of MS6021 raised in rabbit. The small peptide and polyclonal antibody were produced by GL Biochem (Shanghai) Ltd. A protein blot was performed using the Super Signal West Femto Maximum Sensitivity Substrate Kit (THERMO).

### Subcellular localization of MS6021

For the subcellular localization analysis, *MS6021CDS* and *MS6021*Δ*NCDS* (putative signal peptide-coding region deleted) were cloned into the pJIT-163-GFP plasmid. These constructs, as well as the empty plasmid, were introduced into maize protoplasts isolated from delicate leaves, by polyethylene glycol-mediated transformation^57^. The fluorescence signal were examined under a laser scanning confocal microscope (ZEISS LSM 710 NLO).

### Phylogenetic analysis

The protein sequences of the FAR family members from *Arabidopsis*, rice and maize were aligned using the BioEdit tool. The alignment result was used to construct a neighbor-joining polygenetic tree in MEGA 6 (http://megasoftware.net/) using the following parameters: Poisson model, complete deletion, and 1,000 bootstrap replicates.

### *Arabidopsis* transformation

To confirm the functional conservation between MS2 and MS6021, we cloned the *MS2* promoter sequence from Columbia ecotype *Arabidopsis*, *MS6021CDS*, and *MS6021*Δ*NCDS*. These fragments were subcloned into the binary vector pCAMBIA1300 to generate Pro*MS2:MS6021CDS* and Pro*MS2:MS6021*Δ*NCDS*. These constructs were introduced into *Agrobacterium tumefaciens* GV3101 and transformed into the *ms2* mutant plants by the floral dipping method^58^. The transformed seeds were screened using 1/2 plant MS medium containing 20 mg/L hygromycin.

### Transcriptome analysis

Triplicate individuals of uninucleate-stage anthers were harvested from wild-type and *ms6021* plants and were used to extract total RNA with the RNeasy Plant mini Kit (QIAGEN) according to the manufacturer’s instructions. Libraries were constructed in accordance with standard Illumina TruSeq instructions and sequenced using an Illumina Genome Analyzer (Hiseq. 2500; Illumina). The raw reads were filtered to obtain high-quality clean reads and mapped to the maize reference genome (AGPv3; MaizeSequence.org) using TopHat2^59^ with default parameters. The gene expression level was calculated by RPKM^60^. The edgeR package^61^ was used to detect differentially expressed genes (DEGs), which were defined according to the following criteria: more than a two-fold change and FDR less than 0.05. Gene ontology (GO) enrichment and KEGG pathway enrichment analyses were performed using the Bioconductor tool^62^.

## Electronic supplementary material

Supplementary Information

## References

[1] Scott RJ, Spielman M, Dickinson HG (2004). Stamen structure and function. Plant Cell.

[2] Ma H (2005). Molecular genetic analyses of microsporogenesis and microgametogenesis in flowering plants. Annu. Rev. Plant Biol..

[3] McCormick S (1993). Male gametophyte development. Plant cell.

[4] Zhang D, Yang L (2014). Specification of tapetum and microsporocyte cells within the anther. Curr. Opin. Plant Biol..

[5] Goldberg RB, Beals TP, Sanders PM (1993). Anther development: basic principles and practical applications. Plant Cell.

[6] Li N (2006). The rice tapetum degeneration retardation gene is required for tapetum degradation and anther development. Plant Cell.

[7] Li H (2010). Cytochrome P450 family member CYP704B2 catalyzes the {omega}-hydroxylation of fatty acids and is required for anther cutin biosynthesis and pollen exine formation in rice. Plant Cell.

[8] Ariizumi T, Toriyama K (2011). Genetic regulation of sporopollenin synthesis and pollen exine development. Annu. Rev. Plant Biol..

[9] Kunst L, Samuels AL (2003). Biosynthesis and secretion of plant cuticular wax. Prog. Lipid Res..

[10] Jung KH (2006). Wax-deficient anther1 is involved in cuticle and wax production in rice anther walls and is required for pollen development. Plant Cell.

[11] Yeats TH, Rose JK (2013). The formation and function of plant cuticles. Plant Physiol..

[12] Heredia A (2003). Biophysical and biochemical characteristics of cutin, a plant barrier biopolymer. Biochim. Biophys. Acta..

[13] Ahlers, F., Lambert, J. & Wiermann, R. Acetylation and silylation of piperidine solubilized sporopollenin from pollen of typha angustifolia L. *Zeitschrift für Naturforsch. C* **58**, 10.1515/znc-2003-11-1210 (2003). 10.1515/znc-2003-11-1210 14713155

[14] Wallace S, Fleming A, Wellman CH, Beerling DJ (2011). Evolutionary development of the plant and spore wall. AoB Plants.

[15] Blackmore S, Wortley AH, Skvarla JJ, Rowley JR (2007). Pollen wall development in flowering plants. New Phytol..

[16] Wallace S (2015). Conservation of Male Sterility 2 function during spore and pollen wall development supports an evolutionarily early recruitment of a core component in the sporopollenin biosynthetic pathway. New Phytol..

[17] Wilson ZA, Zhang DB (2009). From Arabidopsis to rice: pathways in pollen development. J. Exp. Bot..

[18] Chen W (2011). Male Sterile2 encodes a plastid-localized fatty acyl carrier protein reductase required for pollen exine development in Arabidopsis. Plant Physiol..

[19] Shi J (2011). Defective pollen wall is required for anther and microspore development in rice and encodes a fatty acyl carrier protein reductase. Plant Cell.

[20] Yang X (2014). Rice CYP703A3, a cytochrome P450 hydroxylase, is essential for development of anther cuticle and pollen exine. J. Integr. Plant Biol..

[21] Morant M (2007). CYP703 is an ancient cytochrome P450 in land plants catalyzing in-chain hydroxylation of lauric acid to provide building blocks for sporopollenin synthesis in pollen. Plant Cell.

[22] Dobritsa AA (2009). CYP704B1 is a long-chain fatty acid omega-hydroxylase essential for sporopollenin synthesis in pollen of Arabidopsis. Plant Physiol..

[23] Shi J, Cui M, Yang L, Kim YJ, Zhang D (2015). Genetic and biochemical mechanisms of pollen wall development. Trends Plant Sci..

[24] Azevedo Souza C (2009). A novel fatty Acyl-CoA Synthetase is required for pollen development and sporopollenin biosynthesis in Arabidopsis. Plant Cell.

[25] Dobritsa AA (2010). LAP5 and LAP6 encode anther-specific proteins with similarity to chalcone synthase essential for pollen exine development in Arabidopsis. Plant Physiol..

[26] Kim SS (2010). LAP6/POLYKETIDE SYNTHASE A and LAP5/POLYKETIDE SYNTHASE B encode hydroxyalkyl alpha-pyrone synthases required for pollen development and sporopollenin biosynthesis in Arabidopsis thaliana. Plant cell.

[27] Tang LK, Chu H, Yip WK, Yeung EC, Lo C (2009). An anther-specific dihydroflavonol 4-reductase-like gene (DRL1) is essential for male fertility in Arabidopsis. New phytol..

[28] Grienenberger E (2010). Analysis of TETRAKETIDE alpha-PYRONE REDUCTASE function in Arabidopsis thaliana reveals a previously unknown, but conserved, biochemical pathway in sporopollenin monomer biosynthesis. Plant Cell.

[29] Quilichini TD, Samuels AL, Douglas CJ (2014). ABCG26-mediated polyketide trafficking and hydroxycinnamoyl spermidines contribute to pollen wall exine formation in arabidopsis. Plant Cell.

[30] Wu L (2014). OsABCG15 encodes a membrane protein that plays an important role in anther cuticle and pollen exine formation in rice. Plant Cell Rep..

[31] Zhu L, Shi J, Zhao G, Zhang D, Liang W (2013). Post-meiotic deficientanther1 (PDA1) encodes an ABC transporter required for the development of anther cuticle and pollen exine in rice. J. Plant Biol..

[32] Choi H (2011). An ABCG/WBC-type ABC transporter is essential for transport of sporopollenin precursors for exine formation in developing pollen. Plant J..

[33] Zhang D (2010). OsC6, encoding a lipid transfer protein, is required for postmeiotic anther development in rice. Plant Physiol..

[34] Djukanovic V (2013). Male-sterile maize plants produced by targeted mutagenesis of the cytochrome P450-like gene (MS26) using a re-designed I-CreI homing endonuclease. Plant J..

[35] Wu Y (2016). Development of a novel recessive genetic male sterility system for hybrid seed production in maize and other cross-pollinating crops. Plant Biotechnol. J..

[36] Chen X (2016). IRREGULAR POLLEN EXINE1 is a novel factor in anther cuticle and pollen exine formation. Plant Physiol..

[37] Somaratne Y (2017). *ABNORMAL POLLEN VACUOLATION1* (*APV1*) is required for male fertility by contributing to anther cuticle and pollen exine formation in maize. Plant J..

[38] Zhang D, Wilson ZA (2009). Stamen specification and anther development in rice. Chinese Sci. Bull..

[39] Bonaventure G, Beisson F, Ohlrogge J, Pollard M (2004). Analysis of the aliphatic monomer composition of polyesters associated with Arabidopsis epidermis: occurrence of octadeca-cis-6, cis-9-diene-1,18-dioate as the major component. Plant J..

[40] Franke R (2005). Apoplastic polyesters in Arabidopsis surface tissues–a typical suberin and a particular cutin. Phytochemistry.

[41] Doan TT (2012). Biochemical characterization of a chloroplast localized fatty acid reductase from Arabidopsis thaliana. Biophys. Acta.

[42] Schnable PS (2009). The B73 maize genome: complexity, diversity, and dynamics. Science.

[43] Guilford WJ, Schneider DM, Labovitz J, Opella SJ (1988). High resolution solid state (13)C NMR spectroscopy of sporopollenins from different plant taxa. Plant Physiol..

[44] Qin P (2013). ABCG15 encodes an ABC transporter protein, and is essential for post-meiotic anther and pollen exine development in rice. Plant Cell Physiol..

[45] Zhao G (2015). Two ATP binding cassette G transporters, rice ATP Binding Cassette G26 and ATP Binding Cassette G15, collaboratively regulate rice male reproduction. Plant Physiol..

[46] Rozema J (2001). UV-B absorbance and UV-B absorbing compounds (para-coumaric acid) in pollen and sporopollenin: the perspective to track historic UV-B levels. J. Photochem. Photobiol. B.

[47] Descolas-Gros C, Scholzel C (2007). Stable isotope ratios of carbon and nitrogen in pollen grains in order to characterize plant functional groups and photosynthetic pathway types. New Phytol..

[48] Hahlbrock K, Scheel D (1989). Physiology and molecular biology of phenylpropanoid metabolism. Ann. Rev. Plant Physiol. Plant Mol. Biol..

[49] Edward H. C, McCormic SM, Modena SA (1981). White pollen in mazie. J. Hered..

[50] Napoli CA, Fahy D, Wang HY, Taylor LP (1999). White anther: a petunia mutant that abolishes pollen flavonol accumulation, induces male sterility, and is complemented by a chalcone synthase transgene. Plant Physiol..

[51] Fischer R, Budde I, Hain R (1997). Stilbene synthase gene expression causea changes in flower colour and male sterility in tobacco. Plant J..

[52] Watkins JM, Hechler PJ, Muday GK (2014). Ethylene-induced flavonol accumulation in guard cells suppresses reactive oxygen species and moderates stomatal aperture. Plant Physiol..

[53] Li Y, Kim JI, Pysh L, Chapple C (2015). Four isoforms of Arabidopsis 4-Coumarate:CoA Ligase have overlapping yet distinct roles in phenylpropanoid metabolism. Plant Physiol..

[54] Yadav V (2014). ABCG transporters are required for suberin and pollen wall extracellular barriers in Arabidopsis. Plant Cell.

[55] Quilichini TD, Grienenberger E, Douglas CJ (2015). The biosynthesis, composition and assembly of the outer pollen wall: A tough case to crack. Phytochemistry.

[56] Ding L (2015). HANABA TARANU (HAN) bridges meristem and organ primordia boundaries through PINHEAD, JAGGED, BLADE-ON-PETIOLE2 and CYTOKININ OXIDASE 3 during flower development in Arabidopsis. PLoS Genet..

[57] Yoo S-D, Cho Y-H, Sheen J (2007). Arabidopsis mesophyll protoplasts: a versatile cell system for transient gene expression analysis. Nat. Protocols.

[58] Zhang X, Henriques R, Lin SS, Niu QW, Chua NH (2006). Agrobacterium-mediated transformation of Arabidopsis thaliana using the floral dip method. Nat. Protocols.

[59] Trapnell C, Pachter L, Salzberg SL (2009). TopHat: discovering splice junctions with RNA-Seq. Bioinformatics (Oxford, England).

[60] Ali Mortazavi BAW, McCue K, Schaeffer L (2008). Barbara Wold. Mapping and quantifying mammalian transcriptomes by RNA-Seq. Nat. Methods.

[61] Anders S (2013). Count-based differential expression analysis of RNA sequencing data using R and Bioconductor. Nat. Protocols.

[62] Callahan BJ, Sankaran K, Fukuyama JA, McMurdie PJ, Holmes SP (2016). Bioconductor Workflow for Microbiome Data Analysis: from raw reads to community analyses. F1000Research.

