# Supplementary material for: MALE STERILE6021 (MS6021) is required for the development of anther cuticle and pollen exine in maize
Source: Sci Rep. 2017 Dec 1;7:16736. doi: 10.1038/s41598-017-16930-0 (PMC5711870; doi:10.1038/s41598-017-16930-0)
Supplement: Supplementary file 1 — Supplementary Information [file 41598_2017_16930_MOESM1_ESM.doc]

***Supplementary Material***

***MALE STERILE6021* (*MS6021*) is Required for the Development of Anther Cuticle and Pollen Exine in Maize**

Youhui Tian^1^, Senlin Xiao^1^, Juan Liu^1^, Yamuna Somaratne^1^, Hua Zhang^1^, Mingming Wang^1^, Huairen Zhang^1^, Li Zhao^1^, Huabang Chen^1^*

*Corresponding author

E-mail address: [hbchen@genetics.ac.cn](mailto:hbchen@genetics.ac.cn)


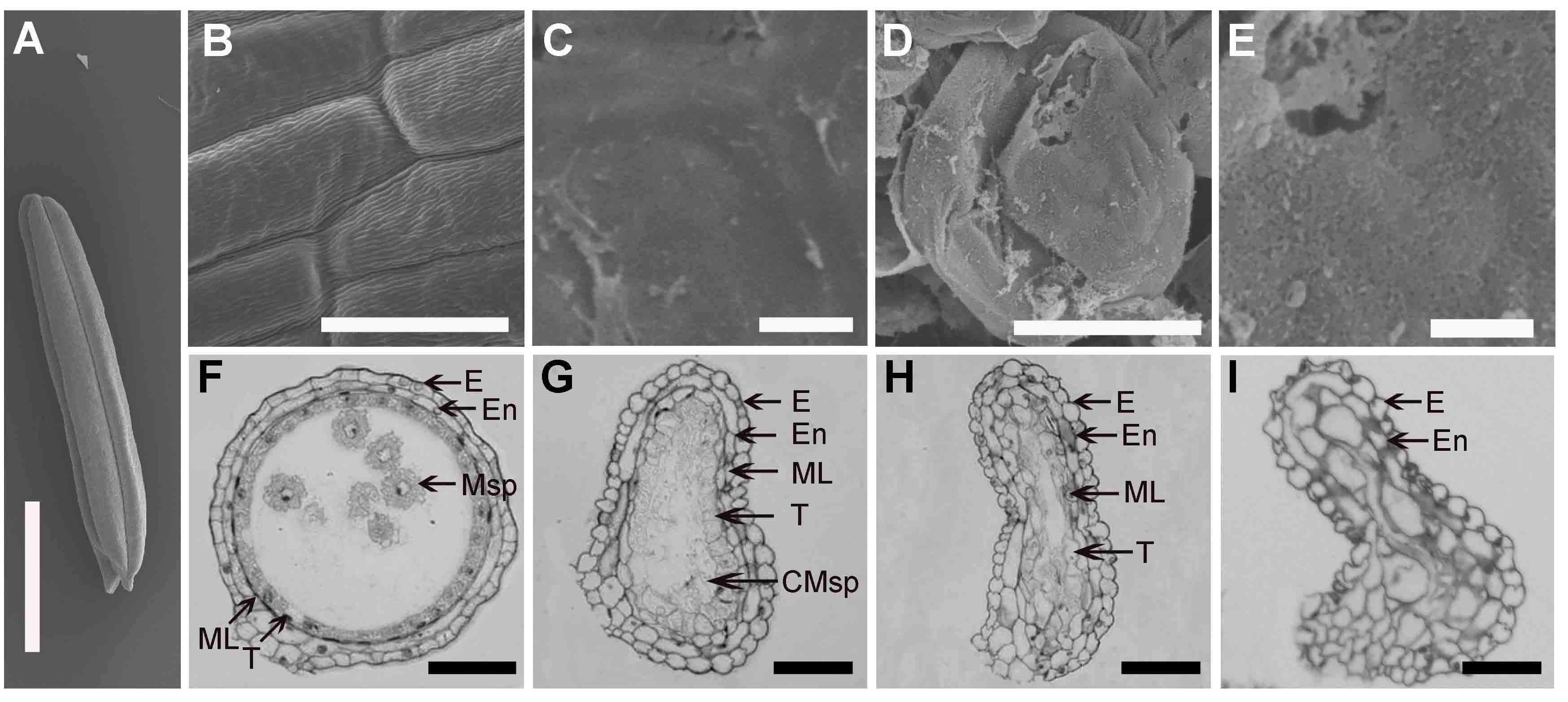
**Supplementary Figures**

**Supplementary**
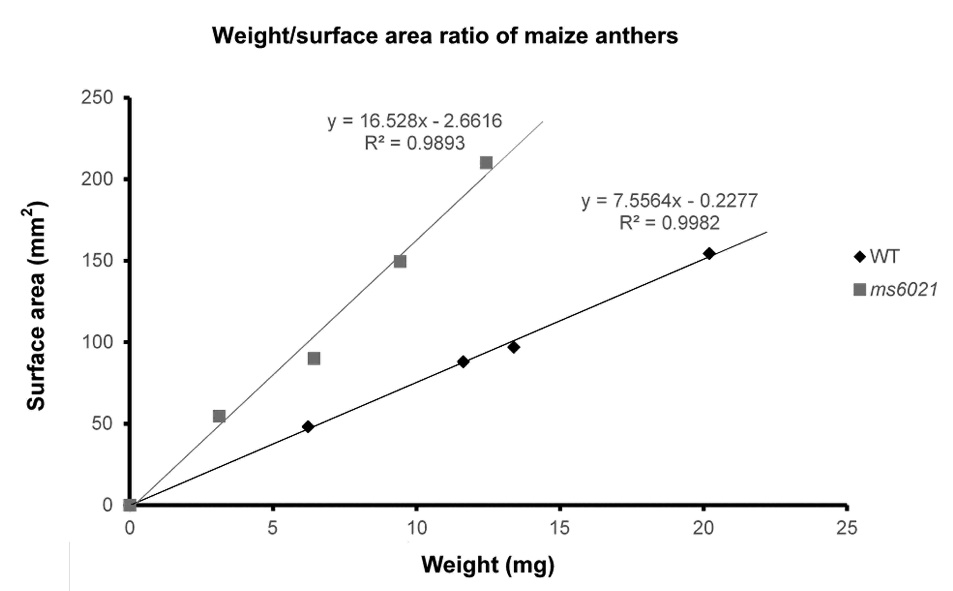
 **Figure S1.** The defective anther surface and pollen exine of *ms6047* mutant. **(**A) to (E) SEM analysis of the anther (A), anther epidermal surface (B), anther wall inner surface (C), pollen (D), and pollen surface (E) of the *ms6047* mutant at mature pollen stage. (F) to (I) Cytological analysis of anther development of the *ms6047* at uninucleate stage (F), large vacuole stage (G), binucleate stage (H), and mature pollen stage (I). CMsp, collapsed microspore; E, epidermis; En, endothecium; ML, middle layer; Msp, microspore; T, tapetum. Bars = 1mm in (A), 20 µm in (B) to (C), 5 µm in (E), and 50 µm in (F) to (I).

**Supplementary Figure S2.** Weight/surface area ratio of wild-type and the *ms6021* anthers.

**Supplementary**
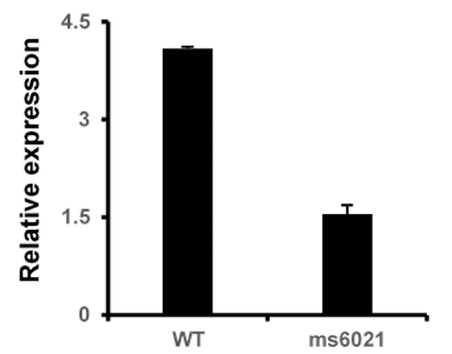
 **Figure S3.** Comparison of *MS6021* expression level between wild-type and *ms6021* at uninucleate stage.

**Supplementary Figure S4.**
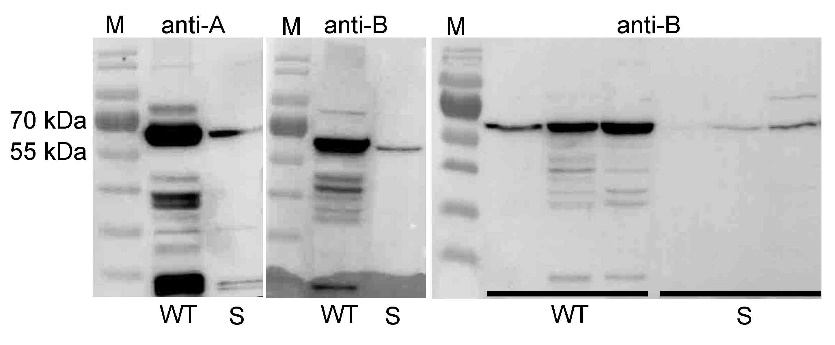
Protein gel blot analysis. Two small peptides were synthesized to generate polyclonal antibodies of MS6021. According to the pre-experiment, anti-B was more specific than anti-A. Hence, anti-B was used in the following western blot assay.


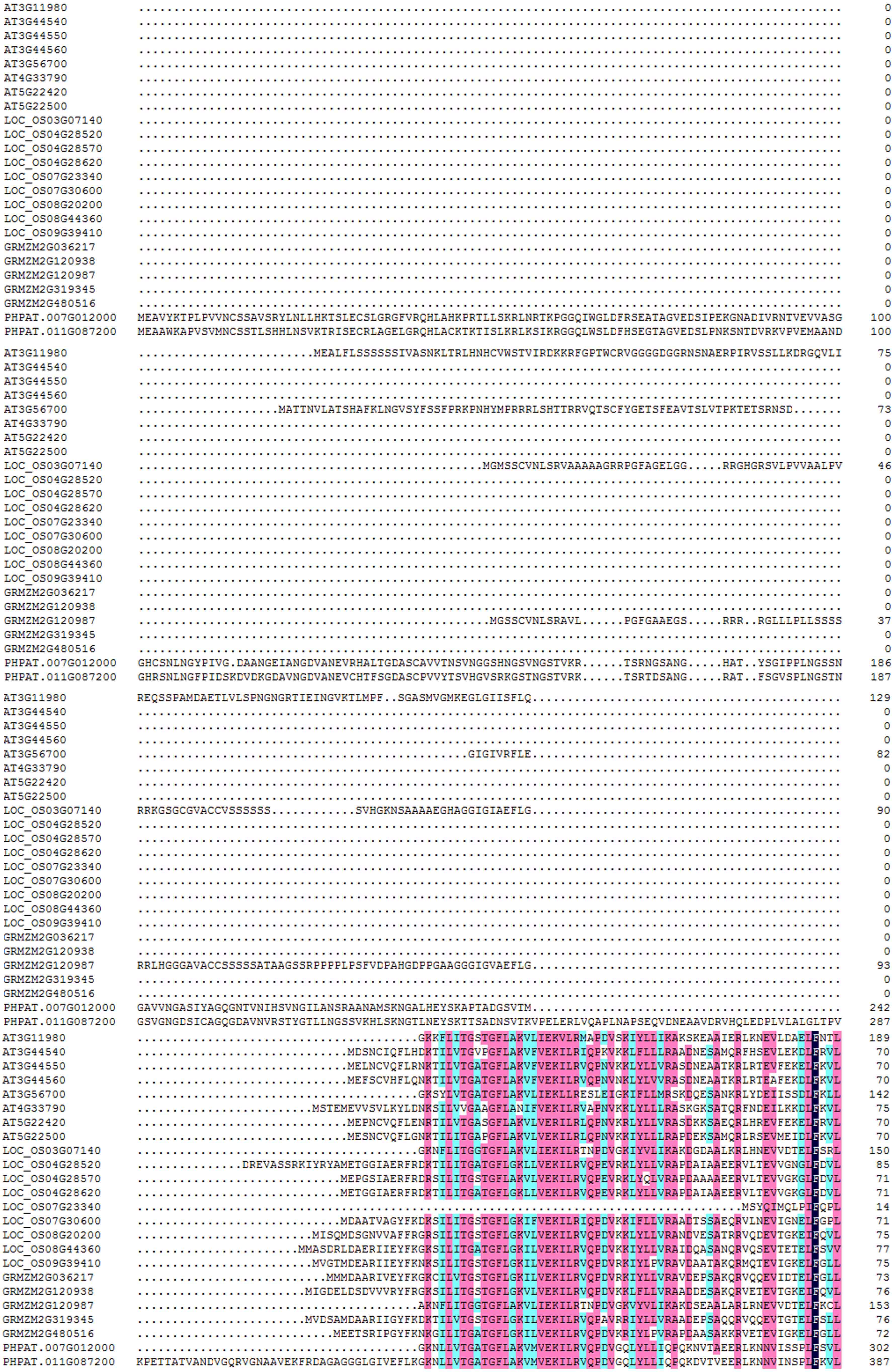


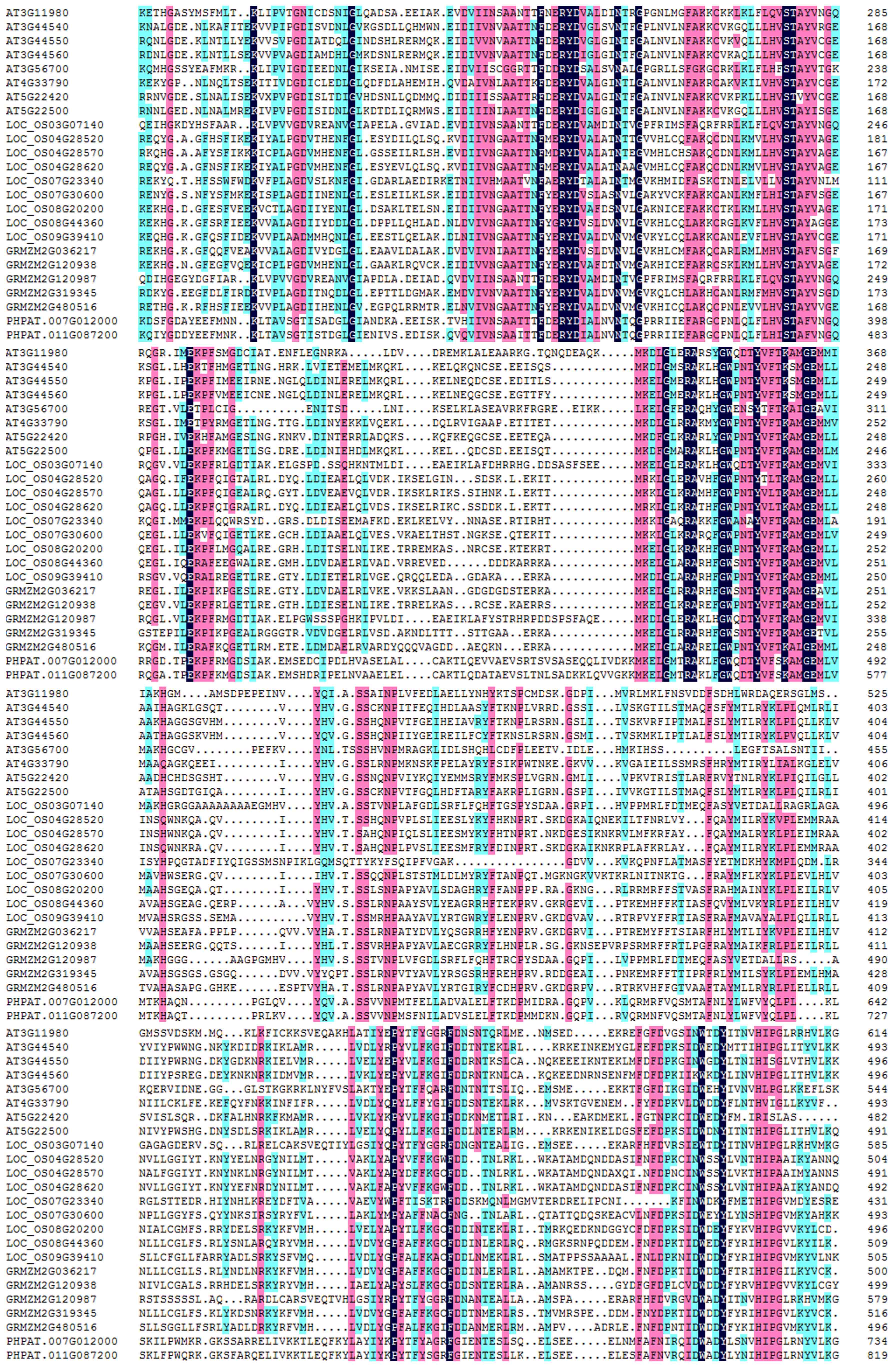


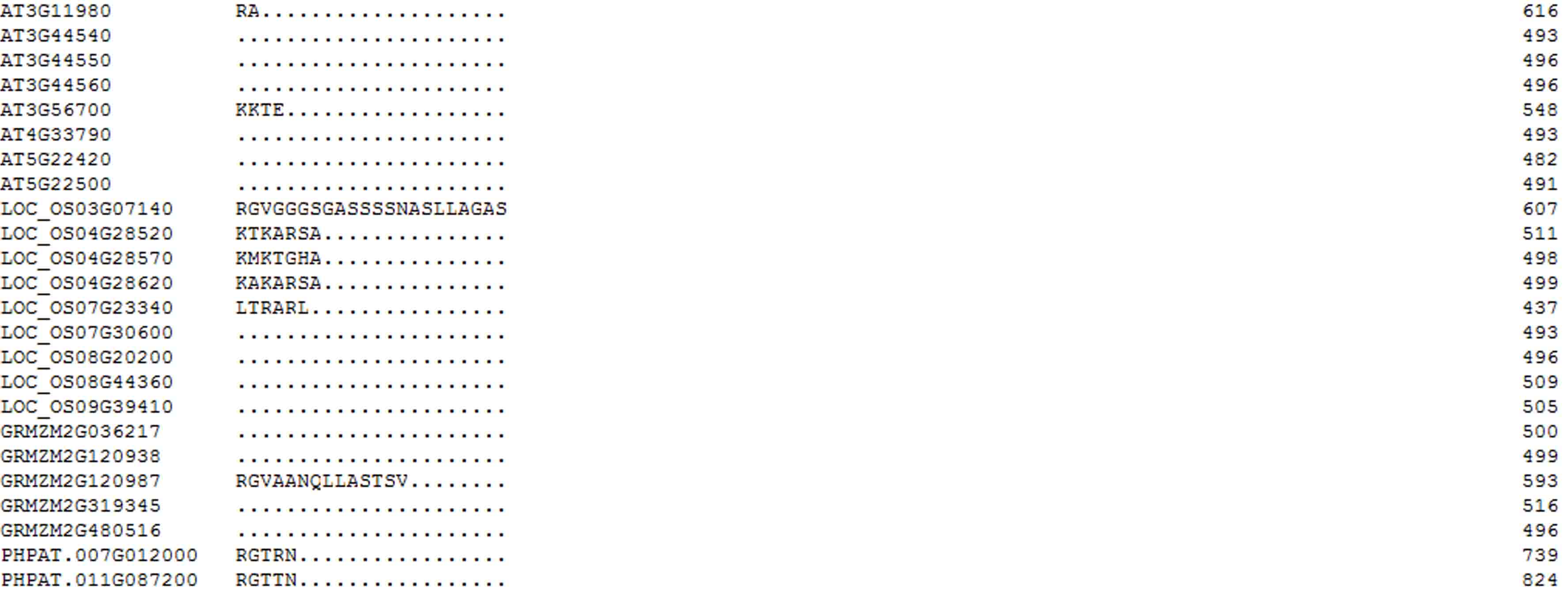
**Supplementary Figure S5.** Sequence alignment of FAR members in *Arabidopsis*, rice and maize.

**Supplementary**
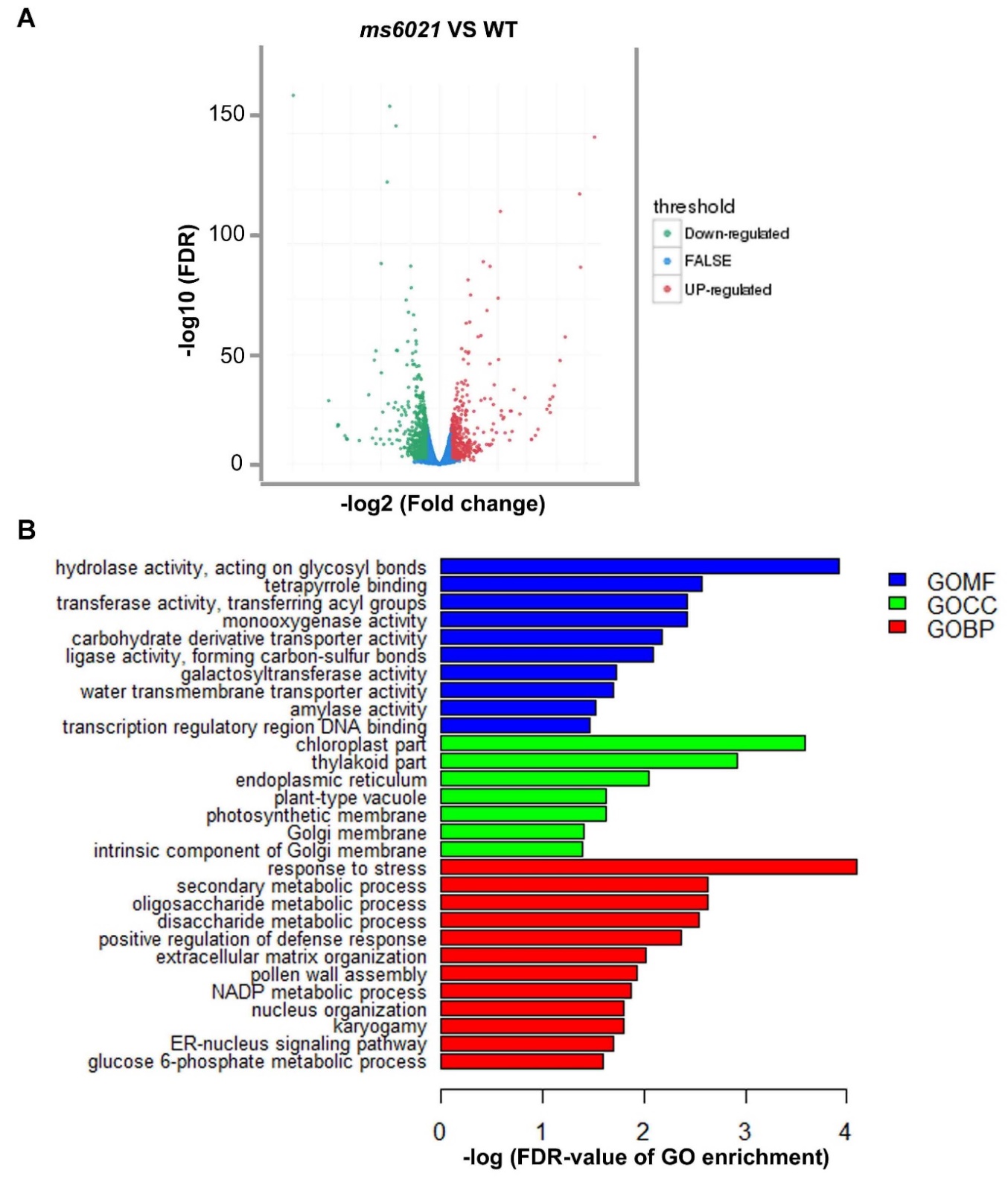
 **Figure S6.** Transcriptome analysis of anther gene expression (*ms6021*/WT) at uninucleate stage. (A) Volcano plot of differentially expressed genes (DEGs). (B) Gene ontology (GO) analysis of DEGs.

**Supplementary Figure S7.**
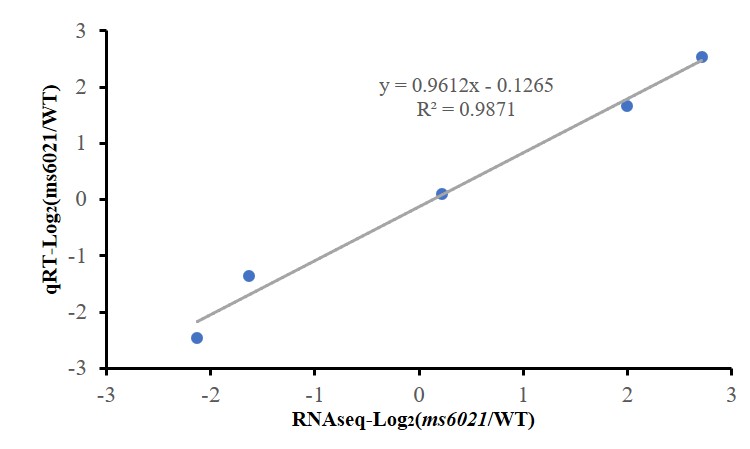
The correlation between RNA-seq data (log2) and qRT-PCR data (log2)

**Supplementary Table S1.** Allelic test analysis

| Testing groups | Fertility | Sterility | χ2 | P |
| --- | --- | --- | --- | --- |
| *ms6022* (**♀**) / *ms6021* (**♂**) | 109 | 91 | 0.81 | > 0.05 |
| *ms6046* (**♀**) / *ms6021* (**♂**) | 142 | 152 | 0.17 | > 0.05 |
| *ms6047* (**♀**) / *ms6021* (**♂**) | 69 | 53 | 1.05 | > 0.05 |

**Supplementary Table S2.** Detailed wax composition in wild-type and *ms6021* anthers

| Wax constituents | Wide Type *ms6021* | | Down |
| --- | --- | --- | --- |
|  | Mean ± SD (ng/mm^2^) | |  |
| C23:0 Alkanes | 0.033 ± 0.009 | 0.026 ± 0.009 | 21.21% |
| C25:0 Alkanes | 6.886 ± 0.897 | 3.885 ± 0.362 | 43.58% |
| C27:0 Alkanes | 16.941 ± 1.080 | 8.826 ± 1.122 | 47.90% |
| C29:0 Alkanes | 0.414 ± 0.019 | 0.175 ± 0.015 | 57.73% |
| C31:0 Alkanes | 6.303 ± 1.117 | 3.072 ± 0.477 | 51.26% |
| C33:0 Alkanes | 1.400 ± 0.546 | 0.713 ± 0.253 | 49.07% |
| C24:0 OH | 0.084 ± 0.012 | 0.059 ± 0.007 | 29.76% |
| C26:0 OH | 0.427 ± 0.195 | 0.344 ± 0.146 | 19.44% |
| C28:0 OH | 0.087 ± 0.018 | 0.090 ± 0.012 | -3.45% |
| Total | 32.575 ± 2.531 | 17.190 ± 2.151 | 47.23% |

**Supplementary Table S3.** Detailed cutin composition in wild-type and *ms6021* anthers

| Cutin constituents | Wide Type *ms6021* | | Down |
| --- | --- | --- | --- |
|  | Mean ± SD (ng/mm^2^) | |  |
| C16:0 acid | 75.836 ± 22.056 | 8.261 ± 2.920 | 89.11% |
| C17:0 acid | 13.043 ± 0.088 | 9.055 ± 3.495 | 30.58% |
| C18:0 acid | 7.161 ± 2.877 | 4.739 ± 0.960 | 33.82% |
| C18:1 acid | 3.158 ± 1.417 | 0.869 ± 0.199 | 72.48% |
| C18:2 acid | 43.919 ± 12.345 | 8.964 ± 3.668 | 79.59% |
| C18:3 acid | 39.311 ± 13.593 | 0.951 ± 0.477 | 97.58% |
| C16:0 diacid | 4.205 ± 2.129 | 1.104 ± 0.144 | 73.73% |
| C18:0 2-OH acid | 2.873 ± 0.353 | 0.585 ± 0.195 | 79.64% |
| C20:0 acid | 7.412 ± 4.284 | 5.502 ± 2.269 | 25.77% |
| C18:0 di-OH acid | 25.879 ± 8.264 | 5.550 ± 1.460 | 78.55% |
| C18:1 18-OH acid | 35.095 ± 7.651 | 8.222 ± 1.465 | 76.57% |
| C18:2 acid | 1.568 ± 0.889 | 0.605 ± 0.120 | 61.45% |
| C18:0 di-OH diacid | 126.959 ± 26.137 | 13.863 ± 2.125 | 89.08% |
| C18:2 18-OH acid | 8.969 ± 1.798 | 2.224 ± 0.236 | 75.21% |
| C22:0 acid | 3.821 ± 1.959 | 3.368 ± 1.087 | 11.86% |
| C22:0 2-OH acid | 12.941 ± 1.509 | 9.858 ± 1.186 | 23.82% |
| C24:0 acid | 2.609 ± 0.919 | 4.513 ±1.765 | -73.00% |
| C24:0 2-OH acid | 0.856 ± 0.389 | 1.435 ± 0.829 | -67.65% |
| C26:0 acid | 0 | 2.481 ± 1.196 | -- |
| C26:0 2-OH acid | 0.873 ± 0.184 | 0.851 ± 0.111 | 2.52% |
| Total | 419.484 ± 25.422 | 92.929 ± 2.151 | 77.85% |

**Supplementary Table S4.** DEGs involved in maize anther development

| **Gene ID** | **Gene Description** | **Log_2_(*ms6021*/WT)** |
| --- | --- | --- |
| GRMZM2G017557 | Flavone and flavanols biosynthesis | 2.403 |
| GRMZM2G165390 | Flavone and flavanols biosynthesis | 1.172 |
| GRMZM2G034471 | Flavone and flavanols biosynthesis | 2.034 |
| GRMZM2G042865 | Flavone and flavanols biosynthesis | 2.403 |
| GRMZM2G074631 | Flavone and flavanols biosynthesis | 1.956 |
| GRMZM2G085812 | Flavone and flavanols biosynthesis | 1.202 |
| GRMZM2G167336 | Flavone and flavanols biosynthesis | 1.882 |
| GRMZM2G151227 | Flavonoid biosynthesis | 2.541 |
| GRMZM2G422750 | Flavonoid biosynthesis | 1.995 |
| GRMZM2G034471 | Flavonoid biosynthesis | 2.034 |
| GRMZM2G380650 | Flavonoid biosynthesis | 1.431 |
| GRMZM2G075513 | Flavonoid biosynthesis | 1.298 |
| GRMZM2G026930 | Flavonoid biosynthesis | 1.436 |
| GRMZM2G167336 | Flavonoid biosynthesis | 1.882 |
| GRMZM2G346095 | Flavonoid biosynthesis | 2.889 |
| GRMZM2G085812 | Flavonoid biosynthesis | 1.202 |
| GRMZM2G108894 | Flavonoid biosynthesis | 2.541 |
| GRMZM2G167336 | Isoflavonoid biosynthesis | 1.882 |
| GRMZM2G096412 | Isoflavonoid biosynthesis | 1.390 |
| GRMZM2G049675 | Isoflavonoid biosynthesis | 1.149 |
| GRMZM2G156310 | Isoflavonoid biosynthesis | -1.101 |
| GRMZM2G075513 | Phenylpropanoid biosynthesis | 1.298 |
| GRMZM2G067096 | Phenylpropanoid biosynthesis | 1.364 |
| GRMZM2G014651 | Phenylpropanoid biosynthesis | 1.258 |
| GRMZM2G110616 | Phenylpropanoid biosynthesis | 1.808 |
| GRMZM2G012236 | Phenylpropanoid biosynthesis | 1.864 |
| GRMZM2G054013 | Phenylpropanoid biosynthesis | 1.025 |
| GRMZM2G074759 | Phenylpropanoid biosynthesis | 1.079 |
| GRMZM2G441347 | Phenylpropanoid biosynthesis | 1.010 |
| AC234526.1 | Phenylpropanoid biosynthesis | 2.527 |
| GRMZM2G180283 | Phenylpropanoid biosynthesis | 1.065 |
| GRMZM5G828987 | Phenylpropanoid biosynthesis | -1.042 |
| GRMZM2G167613 | Phenylpropanoid biosynthesis | -1.160 |
| GRMZM2G110616 | Phenylalanine metabolism | 1.808 |
| GRMZM2G054013 | Phenylalanine metabolism | 1.025 |
| GRMZM2G074759 | Phenylalanine metabolism | 1.079 |
| GRMZM2G441347 | Phenylalanine metabolism | 1.010 |
| GRMZM2G024303 | Fatty acid metabolism | -1.166 |
| GRMZM2G155502 | Fatty acid metabolism | -1.060 |
| GRMZM2G125052 | Fatty acid biosynthesis | 3.529 |
| GRMZM2G156620 | Fatty acid elongation | 1.468 |
| **Gene ID** | **Gene Description** | **Log_2_(*ms6021*/WT)** |
| AC205703.4 | Fatty acid elongation | 1.464 |
| AC205703.4 | Biosynthesis of unsaturated fatty acids | 1.464 |
| GRMZM2G107027 | Cutin, suberine and wax biosynthesis | 1.302 |
| GRMZM2G029912 | Cutin, suberine and wax biosynthesis | 1.211 |
| GRMZM2G434500 | Cutin, suberine and wax biosynthesis | 2.711 |
| GRMZM2G083574 | Cutin, suberine and wax biosynthesis | 1.096 |
| GRMZM2G168416 | ABC transporters | 2.132 |
| GRMZM5G836471 | ABC transporters | 1.115 |
| GRMZM2G054332 | ABC transporters | 1.551 |
| GRMZM2G413774 | ABC transporters | 3.714 |
| GRMZM2G018059 | ABC transporters | 1.147 |
| GRMZM2G127031 | ABC transporters | -1.192 |
| GRMZM2G094490 | ABC transporters | 2.048 |

**Supplementary Table S5.** Expression of genes involved in maize anther development in *apv1*, *ipe1* and *ms6021*

| **Gene ID** | **Arabidopsis/Rice** | **Log_2_(*apv1*/WT)** | **Log_2_(*ipe1*/WT)** | **Log_2_(*ms6021*/WT)** |
| --- | --- | --- | --- | --- |
| GRMZM5G830329(APV1) | CYP703A2/A3 | - | = | = |
| GRMZM2G434500(IPE1) | N/N | - | - | + |
| GRMZM2G120987(MS6021) | MS2/DPW | - | = | - |
| GRMZM2G091822(MS26) | CYP704A1/A2 | - | = | = |
| GRMZM2G307906(MS45) | N/N | = | = | = |
| GRMZM5G890224(ZmMS7) | MS1/PTC1 | = | = | + |
| GRMZM2G108894 | LAP5/N | = | = | + |
| GRMZM2G380650 | LAP6/N | - | = | + |
| GRMZM2G422750 | TT4/N | = | - | + |
| GRMZM2G004683 | TKPR1/N | = | = | + |
| GRMZM2G034360 | N/DPW2 | = | = | = |
| GRMZM2G166671 | N/DTC1 | = | = | = |
| GRMZM2G076526 | ABCG26/ABCG15 | = | = | = |

+, up-regulated; -, down-regulated; =, unaltered expression.

**Supplementary Table S6.** List of primers

| **Primer names** | **Forward Sequences** | **Reverse Sequences** | **Use** |
| --- | --- | --- | --- |
| 2-30 | GCACCAATCCCTTGTACAGC | AAAGCAACTGGTCAAATGGG | Mapping |
| 4-5 | TCCATGTGATTTACGGCTGA | AGCTGTACCAATGGGGAGTG |
| 4-49 | GGTTCTCGACCAATCACTCC | AAGGCGAGGAAAGAGAGAGC |
| 4-89 | CCATGGCGGTTCTGAATCTG | ACCCAGTGTCTTGGGATCTG |
| 3-34 | CTAGGGAGCATTGGTTTGGC | CGACGGGACATAGCTACCAT |
| 2-1 | AAATATCGCCGTCAGAATCG | AAGCTCACCAAGGACCAGG |
| MS2-Promoter | ACAACCAAGAAGATAGCGCACTGGT | GCTTGTTGGTTAAGAAATTGGTGACTTGT | Functional complementation |
| MS6021-CDS | CGTCTTAACACCCACCCACCTACTACTA | GACACTATCAGACAGCCACGATGTTTC |
| MS6021 -Re | GGGGTACCATGGGGAGTTCCTGCGTGAACCTC | CCCAGATCTCACGGAGGTGCTGGCGAGCAGC |
| MS6021ΔN-Re | GGGGTACCATGGACCCGGCGCACGGGGATCC | CCCAGATCTCACGGAGGTGCTGGCGAGCAGC |
| MS6021-SL | CCCAAGCTTATGGGGAGTTCCTGCGTGAACCTC | CGCGGATCCCACGGAGGTGCTGGCGAGCAGC | Subcellular localization |
| MS6021ΔN-SL | CCCAAGCTTATGGACCCGGCGCACGGGGATCC | CGCGGATCCCACGGAGGTGCTGGCGAGCAGC |
| MS6021qRT | TGATGGATCCTGTGGTCCTC | CGGGACCACATCAAGAACAC | qRT-PCR |
| Actin | ACCACAGGTAGCAATAGGTA | CACATTCCAGCAGATGTG |
| MS6021-ISH | GCTGCAAACCGCAACCGCAA | CGTGGTGTTGGCCGCTGAGT | In situ hybridization |
